# Defect Engineering of Mo_2–*x*_CT_*z*_ MXenes through Precursor Alloying and Effects on Electrochemical Properties

**DOI:** 10.1021/acs.chemmater.5c00143

**Published:** 2025-05-30

**Authors:** Rodrigo M. Ronchi, Ningjun Chen, Joseph Halim, Per O. Å. Persson, Johanna Rosen

**Affiliations:** † Materials Design Division, Department of Physics, Chemistry, and Biology (IFM), 4566 Linköping University, SE-581 83 Linköping, Sweden; ‡ Thin Film Physics Division, Department of Physics, Chemistry, and Biology (IFM), Linköping University, SE-581 83 Linköping, Sweden

## Abstract

Defect engineering in the form of the intentional creation of defects has been shown to enhance the properties of two-dimensional materials in various applications. Herein, we systematically explore a simple and reproducible method for introducing random vacancies and pores in Mo-based MXenes by combining first-principles calculations and experiments. The process is based on alloying Mo_2_Ga_2_C with Cr, which is an element that, together with Ga, is selectively etched in hydrofluoric acid, resulting in vacancies and vacancy clusters in the MXene sheets. The limit of Cr incorporation on the metal site was found to be approximately 60 atom % in the precursor powder Mo_2–*x*_Cr_*x*_C. Lower concentrations, up to 25 atom %, were used in the subsequent synthesis of Mo_2–*x*_Cr_*x*_Ga_2_C, since an increasing Cr content promoted the formation of another MAX phase (Mo_2–*x*_Cr_*x*_GaC). A Mo_1.87_CT*_z_* MXene derived from Mo_1.87_Cr_0.13_Ga_2_C (6.5 atom % Cr) exhibited excellent electrochemical behavior, reaching a volumetric capacitance of 1117 Fcm^–3^ at 2 mVs^–1^ scan rate, and suggested that defect concentration can be used to tune the rate capability. Overall, we have demonstrated that using Cr as a sacrificial element in the MAX phase is a simple and effective strategy for the defect engineering of MXenes. Moreover, this method can likely be extended to include other sacrificial elements and MAX phases, making MXene defect engineering a viable pathway for property enhancement across various applications, including energy storage and catalysis.

## Graphical abstract

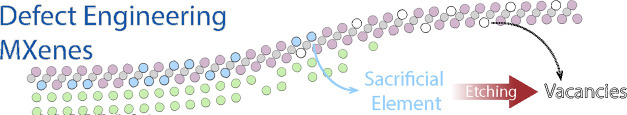

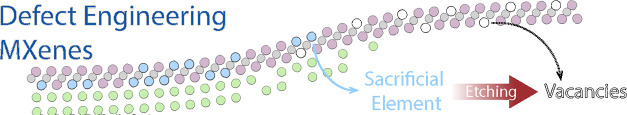

## 1 Introduction

The research on two-dimensional (2D) materials has significantly expanded since the synthesis of graphene,^1^ boron nitride, transition metal dichalcogenides and phosphorene. Exfoliated from their three-dimensional (3D) van der Waals parent phases,^2^ these 2D materials hold great potential for numerous applications, including energy storage, catalysis, gas separation, and water desalination and purification.^2–4^

The synthesis of MXenes in 2011 further boosted the attention of the scientific community.^5^ MXenes are transition metal (M) carbides, nitrides, or carbonitrides (X) with the general formula M_*n*+1_X*_n_* T_z_, where *n* = 1–4 and T_*z*_ refers to surface terminations, such as O, OH, F, Cl, Br, S, Te, and others.^4,6^ They are generally synthesized by selectively etching the A element, such as Al, from the 3D parent MAX phase.^7^ Since the first MXene, Ti_3_C_2_T_*z*_,^5^ the family has expanded to more than 50 members and continues to grow,^6^ driven by their promising results in a wide range of applications, including energy storage, electromagnetic interference shielding, catalysis, water desalination, and biomedicine.^4^

Further development and optimization of 2D materials and their properties would contribute to resolving current environmental challenges.^8^ One possible route is the rational creation of defects, known as defect engineering, which allows tuning of properties such as charge carrier concentration and the activity of adsorption/storage sites.^3,8^ The introduction of pores in both graphene^3^ and MXene^9^ materials has improved their molecular separation and electrochemical energy storage performance. Additionally, due to the introduction of ordered vacancies, Mo_4/3_CT_*z*_ and W_4/3_CT_*z*_ had enhanced performance in tests related to supercapacitors and hydrogen evolution reaction (HER) electrocatalysts, respectively.^10,11^

Several methods have been reported for creating vacancies and pores in MXenes. Partial oxidation of Ti_3_C_2_T_*z*_ sheets, resulting in TiO_2_ particles and their subsequent dissolution with HF, led to the formation of pores.^9^ However, X-ray photoelectron spectroscopy (XPS) analysis revealed an increased TiO_2_ content in the 2D material, likely due to facilitated oxidation and/or incomplete removal of TiO_2_ particles from the preoxidation procedure.^9^ Additionally, a focused electron beam has been used to create nanopores in Ti_3_C_2_T_*z*_ and Ti_2_CT_*z*_.^12^ Recently, this technique was enhanced by combining the beam with simulations and a feedback-controlled scanning position system, enabling selective removal of atoms in a highly controlled manner.^13^

The use of sacrificial alloying elements in MAX phases has facilitated the creation of vacancies and pores in 2D MXenes upon etching. By leveraging the different driving forces for mixing metal atoms, the resulting defects can be either ordered or randomly distributed within the 2D structure. For example, selective etching of Sc and Al atoms from the (Nb_0.67_Sc_0.33_)_2_AlC^14^ MAX phase results in randomly distributed defects in 2D sheets of Nb_1.33_CT_*z*_. In contrast, ordered vacancy MXenes (Mo_4/3_CT_*z*_ and W_4/3_CT_*z*_) are obtained by removing Al and chemically ordered Sc or Y (M″) from quaternary in-plane ordered *i*-MAX phases, such as (Mo_2/3_M″_1/3_)_2_AlC and (W_2/3_M″_1/3_)_2_AlC. Recently,^15^ by taking advantage of the high Cr solubility in Mo_2_C carbides, we synthesized Mo_1.74_CT_*z*_-based MXenes with randomly distributed vacancies and pores by selectively etching the parent Mo_1.74_Cr_0.26_Ga_2_C. Although Mo_2–*x*_Cr_*x*_Ga_2_C is not a MAX phase-like material according to standard definitions,^16^ in this work it will be referred to as a MAX phase. HF-etching not only dissolved Ga, converting the MAX phase to a 2D MXene, but also removed Cr, facilitating the formation of vacancies and pores. This simple method offers an effective approach for creating vacancies and vacancy clusters in MXenes, with relatively good control over defect concentration.^15^

Motivated by the hypothesis that the vacancy fraction in the MXene can be controlled by varying the Cr content, we explore defect engineering by tuning the Cr concentration. Using density functional theory (DFT) combined with materials synthesis and characterization, the maximum Cr solubility in Mo_2–x_Cr_*x*_C was found to be approximately 60 atom %, and a decrease in the yield of Mo_2–*x*_Cr_*x*_Ga_2_C was observed with increasing Cr content. Selective etching of the parent 3D alloy phases resulted in the formation of defects, vacancies, and pores in the 2D sheets. The Mo_1.87_CT_*z*_ MXene, derived from a parent precursor with 6.5 atom % Cr on the metal site exhibited excellent electrochemical properties, showing nearly double the capacitance of its stoichiometric counterpart (Mo_2_CT_*z*_). Overall, defect engineering appears to be a promising and efficient method for tuning the material properties.

## 2 Methods

### 2.1 Computational Details

All DFT^17,18^ calculations were performed using Vienna *Ab initio* simulation package (VASP)^19,20^ within the plane-waves formalism. The Perdew, Burke, and Ernzerhof generalized gradient functional (PBE-GGA)^21^ was used to model the exchange-correlation interactions, while the projector augmented wave (PAW) method^22,23^ was used to describe the electron–ion interaction. The geometry optimizations used Monkhorst–Pack grids^24^ to sample the Brillouin zone, and the plane wave cutoff energy was set to 520 eV.

To model the chemically disordered alloys, the special quasi-random structure (SQS) method was employed.^25^ The *k*-point density, energy, and force convergence parameters were optimized within energy differences of 2 meV/atom. Through size convergence tests with respect to total energy (Figure S1), we used SQSs between 90 and 120 atoms for the different compounds and space groups. Moreover, initial calculations (Table S1) showed that ferromagnetic and nonmagnetic configurations are degenerate, and thus, all calculations were performed considering the nonmagnetic configuration. Antiferromagnetic (AFM) configurations were not evaluated due to challenges in identifying the magnetic ground state in a random SQS cell with chemical disorder on the metal atom sites as well as potential partial disorder in the spin configuration. Further, a previous study^26^ for a similar MAX phase (Cr_2_GaC) showed a maximum energy difference of only 4 meV/atom for AFM configurations, which, for the screening purpose of this paper, can be disregarded.

Phase stability evaluation was based on a linear optimization procedure to identify the set of most competing phases for a given composition.^27–29^ This method has been successfully used to predict the existence of several MAX^16,28^ and MAB^30^ phases, including Mo_2_Ga_2_C,^31^ and can be considered a better stability descriptor than the often-used formation energy. The procedure is based on a comparison of the energy of a given compound (e.g., Mo_1.87_Cr_0.13_Ga_2_C) with the minimum energy of the linear combination of all competing phases in the system, min *E_cp_* (e.g., in the quaternary Mo–Cr–Ga–C system),^27–29^ expressed as

1 $${\Delta H}_{\mathrm{cp}}\left(\mathrm{phase}\right)=E\left(\mathrm{phase}\right)-\min\;E_{\mathrm{cp}}$$

The phase is considered thermodynamically stable when the formation enthalpy (Δ*H*_cp_) is below zero, i.e., when the phase of interest is of an energy lower than the set of most competing phases.^27–29^ The energy of the set of most competing phases, min *E*_cp_, is found by

2 $$\min\;E_{\mathrm{cp}}=\sum_{i}^{n}x_{i}E_{i}$$

where *x*_*i*_ and *E*_*i*_ is the amount and energy of a compound *i* being part of the phase space built up by the constituting elements. The amount *x*_*i*_ of each phase is constrained to match the stoichiometry corresponding to the phase under investigation. The competing phases considered in this paper were acquired from open databases including OQMD,^32,33^ Materials Project^34^ and Springer Materials.^35^

For temperatures *T* ≠ 0 K, the formation enthalpies, Δ*H*_cp_, are replaced by the Gibbs Free energy of formation, Δ*G*_cp_, including the effect of entropy:

3 $${\Delta G}_{\mathrm{cp}}\left(\mathrm{phase}\right)={\Delta H}_{\mathrm{cp}}\left(\mathrm{phase}\right)-T\Delta S$$

The temperature used in this work is set as the experimental synthesis temperature for each compound. Thus, *T* = 1673 K (1400 °C) is used for Mo_2–*x*_Cr_*x*_C and *T* = 1043 K (740 °C) is used for Mo_2–*x*_Cr_*x*_Ga_2_C and Mo_2–*x*_Cr_*x*_GaC. Δ*S* is approximated by the configurational entropy expressed in terms of an ideal solid solution per formula unit, given by

4 $$\Delta S=-mk_{B}\left[y\;\ln\left(y\right)+\left(1-y\right)\ln\left(1-y\right)\right]$$

where *k*_B_ is the Boltzmann constant, *y* is the concentration of the alloying element on the metal site, and *m* is the number of alloying sites. For all systems (Mo_2_C, Mo_2_GaC, and Mo_2_Ga_2_C), *m* = 2 is used for the configurational entropy of the metal atoms (Mo and Cr). For the Mo_2_C­[*P*6_3_ */mmc*] system, an additional entropy term with *m* = 1 is used to represent the 0.5 carbon occupancy and the total entropy is the sum of both contributions (metals and C occupancy). The explicit contribution from temperature-dependent effects such as electronic entropy and lattice vibrations has not been considered, as these contributions tend to cancel out.^36^

Chemical bonding was investigated using the LOBSTER program v5.0.0 ^37^ to generate projected crystal orbital hamiltonian populations (pCOHP).^38–40^ The results are presented as −COHP to maintain the analogy of the crystal orbital overlap population (COOP) analysis. Positive −pCOHP values represent bonding interactions, while negative ones refer to antibonding interactions (nonbonding states have zero −pCOHP). The images were generated with VESTA software.^41^

### 2.2 Experimental Details

#### Synthesis of 3D Binary Carbide

Mo_2–*x*_Cr_*x*_C powders were synthesized through solid-state synthesis using a tube furnace, with Mo (99.95 wt %, 3 to 7 μm, Alfa Aesar), Cr (>99 wt %, <325 mesh, Sigma-Aldrich), and C (99.9995 wt %, 200 mesh, Alfa Aesar) elemental powders as precursors. They were mixed with a mortar and pestle in the different molar ratios evaluated (see Table S7) and annealed at 1400 °C for 25 h under 5 sccm Argon flow (heating and cooling rates of 5 °C/min).

#### Synthesis of 3D Ternary Carbide

The synthesized Mo_2–*x*_Cr_*x*_C powder (above) and elemental Ga (rods, 99.999 wt %, Wuhan Xinrong Bew Material Co. Ltd., Wuhan, China) were mixed in a molar ratio of 1:8. Specifically, after melting Ga rods using a hot water bath, Ga droplets (from a pipet) and Mo_2–*x*_Cr_*x*_C powders were added to an alumina crucible in alternating layers. They were mixed thoroughly with a plastic pipet and heated up to 740 °C (under 5 sccm Ar flow) and kept for 6.5 days. Both heating and cooling rates were 10 °C/min. At least three batches of samples were made for each chromium concentration. The Cr concentrations investigated were 0, 6.25, 13, 19, and 25 atom % Cr (on the metal site). A 6 M hydrochloric acid solution (12M, Fisher Chemicals, Germany) was used to remove excess Ga, and the mixture was washed with deionized (DI) water to achieve pH ≈ 6. Finally, the washed sample was filtered and crushed to achieve a powder.

#### Synthesis of 2D MXene

1.5 g of the synthesized MAX phase was reacted with 25 vol % hydrofluoric acid, HF (50 vol %, Sigma-Aldrich AB, Stockholm), at room temperature (RT) for 5 days under continuous agitation. After washing to pH ≈ 6, tetrabutylammonium hydroxide, TBAOH, (Sigma-Aldrich, 25 vol %, AB, Stockholm, Sweden) was added to intercalate and delaminate the layers following ref 42. The sedimented powder was washed with *N*_2_-deaerated DI water to remove the TBAOH. 40 mL MXene suspension with 3.1 mg/mL was achieved by shaking the MXene mixture for 15 min using a vortex mixer (LSE, Corning Inc., Glendale, AZ). The remaining supernatant of delaminated MXene flakes was collected and the suspension was vacuum filtered, resulting in free-standing films. The detailed synthesis procedure for all materials was in line with previous work.^15^

### 2.3 Materials Characterization

X-ray diffraction, XRD (PANalytical diffractometer, Cu Kα radiation source), was used to probe the crystal structure of the materials. 1/2° and 5 mm divergence and receiving slits were used, and a Nickel filter was added to avoid any *K*_β_ signal. The step size and time per step were 0.0084° and 32 s, respectively. Structural parameters and weight percentages of the different phases were obtained by a Rietveld refinement procedure^43,44^ using the FULLPROF software.^45^ Thompson-Cox-Hastings pseudo-Voigt with an Axial divergence asymmetry function was used to model peak shapes, and instrument parameters were determined by using a NIST LaB_6_ standard material. The refined parameters were scale factors, zero offset, five background parameters, lattice parameters, *Y* profile, asymmetry parameters, overall thermal factor, preferred orientation and atomic positions. The Mo/Cr occupancies were kept as nominal values (i.e., not refined), motivated by the performed materials composition analysis.

Materials morphology and chemical composition was evaluated using scanning electron microscopy, SEM (LEO 1550), combined with an energy dispersive X-ray spectrometer (EDX). At least 10 particles were used to acquire the data for every sample. For the MXene film, two areas of 185 × 185 μm^2^ were used.

To investigate the atomic structure and defects in the MXene single flakes, high-angle annular dark-field scanning transmission electron microscopy (HAADF-STEM) imaging and EDX elemental analysis were performed using the Linköping double-corrected and monochromated FEI Titan^3^ (S)­TEM electron microscope operated at 300 kV.

The electrochemical measurements for a three-electrode Swagelok cell were recorded by using a Biological VSP potentiostat. A free-standing film of (Mo,Cr)_2_CT_*x*_ MXene was used as the working electrode, activated carbon (PTFE 10 wt %) as the counter electrode, and Ag/AgCl as the reference electrode in the three-electrode cell (1 M H_2_SO_4_ electrolyte). The density used to calculate the volumetric capacitance of the MXene film was 3.67 g/cm^3^. Different scan rates and current densities were used to record cyclic voltammetry (CV) and galvanic charge–discharge (GCD) curves. The cyclic stability was measured at a current density of 10 Ag^1–^ and the electrochemical impedance spectroscopy (EIS) data was collected under open-circuit potential in the frequency range from 0.01 to 10^5^ Hz.

## 3 Results and Discussion

Figure 1 schematically represents the synthesis procedure, highlighting the sequential steps involved in creating intentional vacancies in an MXene material. The process begins with the synthesis of a Mo_2–*x*_Cr*_x_* C precursor, which is then transformed into the MAX phase alloy Mo_2–*x*_Cr_*x*_Ga_2_C. Subsequent etching and delamination steps lead to the formation of an MXene containing randomly distributed vacancies and pores, as a result of the targeted removal of Cr atoms from the metal layers.

**Figure 1:**
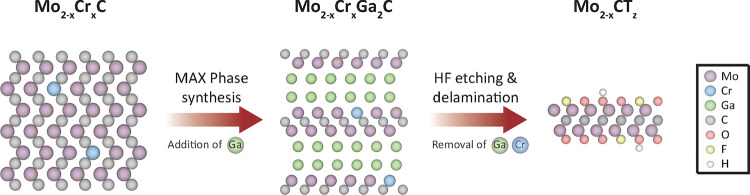
Schematic of the synthesis procedure, starting from the Mo_2–*x*_Cr_*x*_C precursor used in the Mo_2–*x*_Cr_*x*_Ga_2_C MAX phase synthesis, followed by etching and delamination to obtain defect-engineered Mo_2–*x*_CT_*z*_ MXene.

### 3.1 Theoretical Evaluation of Phase Stability

The phase stability was evaluated by calculating the Gibbs free energy of formation. In general, the method considers experimentally known and theoretically predicted competing phases. However, for stable random alloys, if compositions close in phase space are included in the set of competing phases, Δ*G*_cp_ will be close to zero, resembling a so-called convex hull formalism (see Tables S2 and S3, compiled in Figure S2). The Gibbs free energy of formation for a specific alloy composition, such as Mo_0.5_Cr_0.5_Ga_2_C, is therefore calculated considering (i) the end-member constituents (e.g., Mo_2_C, Cr_2_GaC, etc.) and (ii) alloys with the same metal ratio as the alloy phase of interest (Mo_0.5_Cr_0.5_C and Mo_0.5_Cr_0.5_GaC) but neglecting adjacent Cr concentrations.

When evaluating the incorporation of Cr in the 3D Mo_2–*x*_Cr_*x*_C compound, three different stable space groups suggested for Mo_2_C in the literature^46–48^ must be considered, see Figure S3. For temperatures above 1960 °C, a hexagonal L03-type structure (space group *P*6_3_ */mmc*) is formed, where the metal atoms are arranged in a hexagonal closed-packed array and carbon atoms randomly occupy half of the octahedral interstitial sites. Between 1960 and 1350 °C, an ε-Fe_2_N-type structure (space group *P*3̅*m*1) forms, while temperatures below 1350 °C result in a ξ-Fe_2_N-type structure (space group *Pbcn*). To avoid confusion with different notations used in the literature, the space groups will be explicitly indicated in square brackets such as Mo_2_C­[*Pbcn*].

The Gibbs free energy of formation for these Mo_2–*x*_Cr_*x*_C structures is shown in Figure 2a. Regardless of the Cr content, the orthorhombic space group remains the most stable, in agreement with previous theoretical^46,49,50^ and experimental^46–48^ results. Additionally, the effect of configurational entropy can be seen in Figure S2. Neglecting this term suggests that Mo_2_C­[*Pbcn*] is unstable across the entire range of Cr concentrations. When included, the phase becomes stable (or nearly stable) from approximately 0 to 75 at% Cr (with positive Gibbs free energy of formation at Cr_2_C).

**Figure 2:**
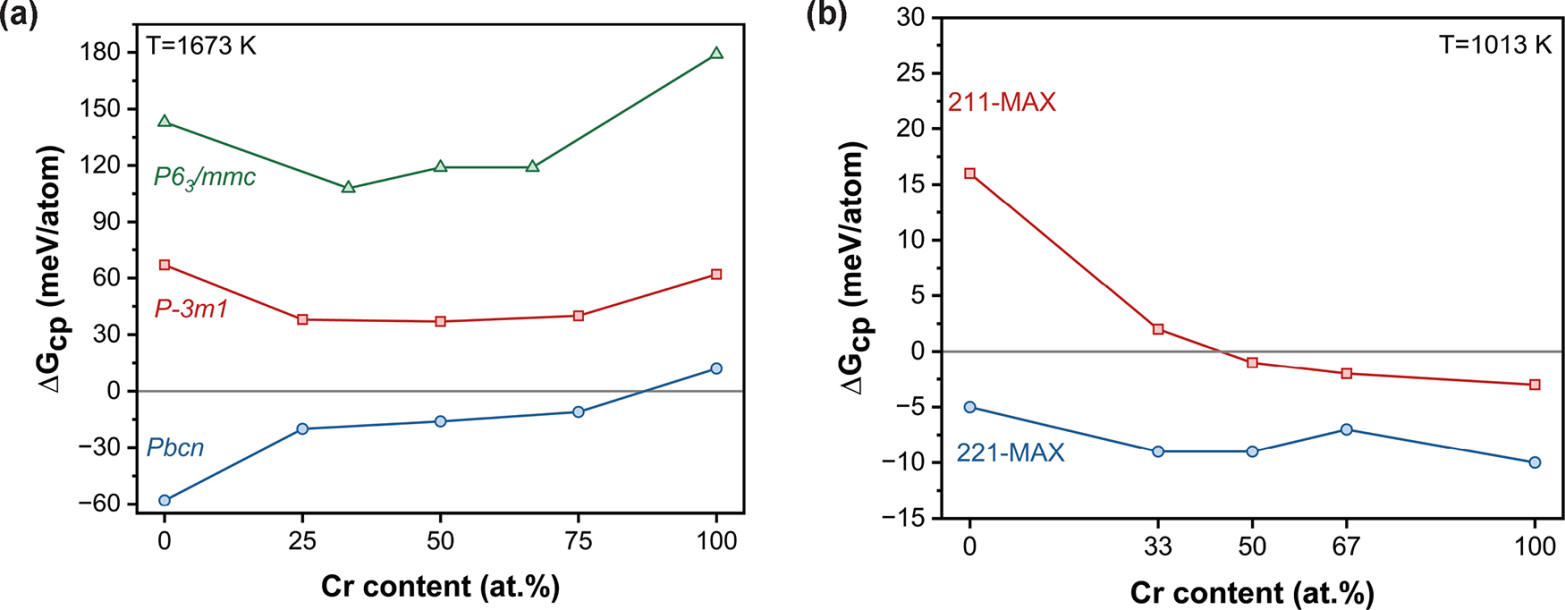
Calculated Gibbs free energy of formation, Δ*G*_cp_, vs Cr content for (a) Mo_2–*x*_Cr_*x*_C and (b) Mo_2–*x*_Cr_*x*_Ga_2_C (221) and Mo_2–*x*_Cr_*x*_GaC (211)­MAX phases (from Tables S4 and S5).

Figure 2b shows the calculated phase stability for the Mo_2–*x*_Cr_*x*_Ga_2_C [*P*6_3_ */mmc*] MAX phase-like material, also referred to as 221-MAX in the following discussions. A full list of competing phases can be found in Table S6. The entire range of Cr alloying is predicted to be stable, even suggesting the possibility of synthesizing a ternary Cr_2_Ga_2_C phase. However, since Mo_2–*x*_Cr_*x*_C is first produced and then reacts with Ga to form the targeted MAX phase, the process is limited by the Cr solubility in the Mo_2–*x*_Cr_*x*_C precursor, which is experimentally indicated to be near 60 atom %.^51^ It is important to note that the Mo_2–*x*_Cr_*x*_GaC MAX phase (referred to as 211-MAX) shows a decrease in the Gibbs free energy of formation with increased Cr concentration, reaching negative values at around 50 atom % Cr at *T* = 1013 K.

Considering the three phases evaluated theoretically, the predicted stable and unstable concentrations diverge slightly from experimental findings: Mo_2–*x*_Cr_*x*_C has a measured maximum solubility around 60 atom % Cr at 1400 °C,^51^ while the 221 phase is not obtained experimentally when 25 atom % Cr is added (see Section 3.3). This difference most likely originates from the computational approximations involved, e.g., ideal composition/stoichiometry, absence of various defects such as vacancies and grain boundaries, and no consideration of reaction kinetics. These approximations may explains why a theoretical value below (above) zero may not allow (suppress) successful experimental synthesis,^36^ also evident from a high-throughput theoretical study showing that 80% of the synthesizable compounds in the ICSD database compounds have an energy above convex hull up to 36 meV per atom.^52^ Nonetheless, our experimental validation is consistent with both trends identified from the computations, namely, that 221-MAX phases can be formed upon Cr addition and with increasing Cr concentrations (i) Mo_2-x_Cr_x_C becomes more unstable and (ii) 211-MAX phase becomes more stable.

### 3.2 Experimental Evaluation of Mo_2_C Alloyed with Cr

The XRD results (Figure S5) show that the 66 atom % Cr sample contains a small amount of Mo_1–*x*_Cr_*x*_C impurities, which significantly increases in the 87.5 atom % Cr sample (C and Cr_3_C_2_ impurities also appear in the latter). This indicates that, under the experimental conditions used, 66 atom % Cr is close to the solubility limit of Cr in Mo_2–*x*_Cr_*x*_C, consistent with previously reported incorporation of at least 60 atom % Cr at 1350 °C.^51^ Up to 66 atom % Cr, all samples exhibited similar XRD spectra, but the peaks shifted toward larger angles, e.g., (121) peak position moves from 39.4 to 40.9° (Figure S5b), due to the incorporation of smaller Cr atoms into the Mo_2–*x*_Cr_*x*_C structure. This shift is also reflected in Figure 3a, which shows a continuous linear decrease in the lattice parameters, as determined through Rietveld analysis (Table S8). Moreover, the simulated lattice parameters are within 1% of the experimentally obtained values, demonstrating the strong agreement between theoretical and experimental results.

**Figure 3:**
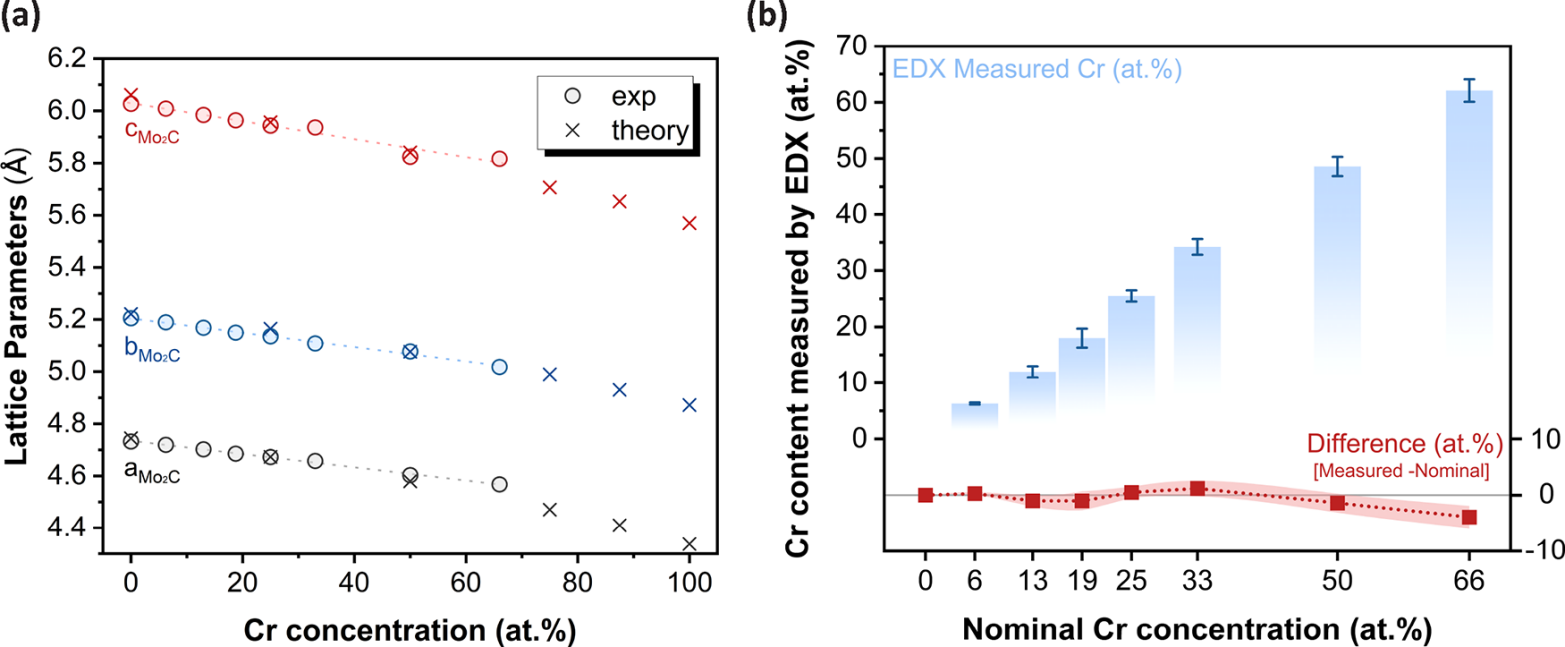
(a) Summary of experimental (circles) and theoretically calculated lattice parameters (‘×’) versus Cr concentration for Mo_2–*x*_Cr_*x*_C­[*Pbcn*]. (b) Measured EDX Cr concentration (bars) and the difference between the measured and nominal Cr concentration (dotted red line) with its associated statistical uncertainty (shaded region) versus the initial nominal Cr concentration used for synthesis.

SEM analysis (Figure S6) shows similar particle morphologies across different Cr contents, while EDX measurements confirm the incorporation of Cr into the Mo_2–*x*_Cr_*x*_C structure. Figure 3b demonstrates that the measured Cr content aligns with the nominal values (targeted composition) within the statistical margin of error. It is worth noting that the targeted 66 atom % Cr sample exhibits a slightly lower Cr concentration (atom 62% on average) and shows a greater deviation in the measured lattice parameters compared to theoretical predictions. These findings further support that this nominal composition exceeds the maximum Cr solubility at this temperature. Due to uncertainties in EDX related to the quantification of light elements, the carbon concentration was not measured and full occupancy of the C sites was assumed.

### 3.3 Synthesis and Characterization of Mo_2_Ga_2_C Alloyed with Cr

Detailed EDX, XRD, and Rietveld evaluations of the Mo_2–*x*_Cr_*x*_Ga_2_C MAX phase synthesized under different conditions are provided in the Supporting Information. From the Rietveld refinement results (Tables S9–13), summarized in Figure 4a, the 221-MAX phase content decreases significantly with increasing Cr concentration, reaching nearly zero for the 25 atom % Cr sample. Attempts to improve phase content by varying the synthesis temperature to 590, 690, and 840 °C (with the same synthesis time) were made, but the highest yield of the 221-MAX phase was obtained at 740 °C (Figure S7). Consequently, the 221-MAX phase yield declines substantially with increasing Cr content, while higher temperatures lead to the predominance of the 211-MAX phase and, eventually, Mo_2–*x*_Cr_*x*_C. This trend was indicated by our calculations (Section 3.1), which showed an increase in the stability of the 211-MAX phase with a higher Cr concentration.

**Figure 4:**
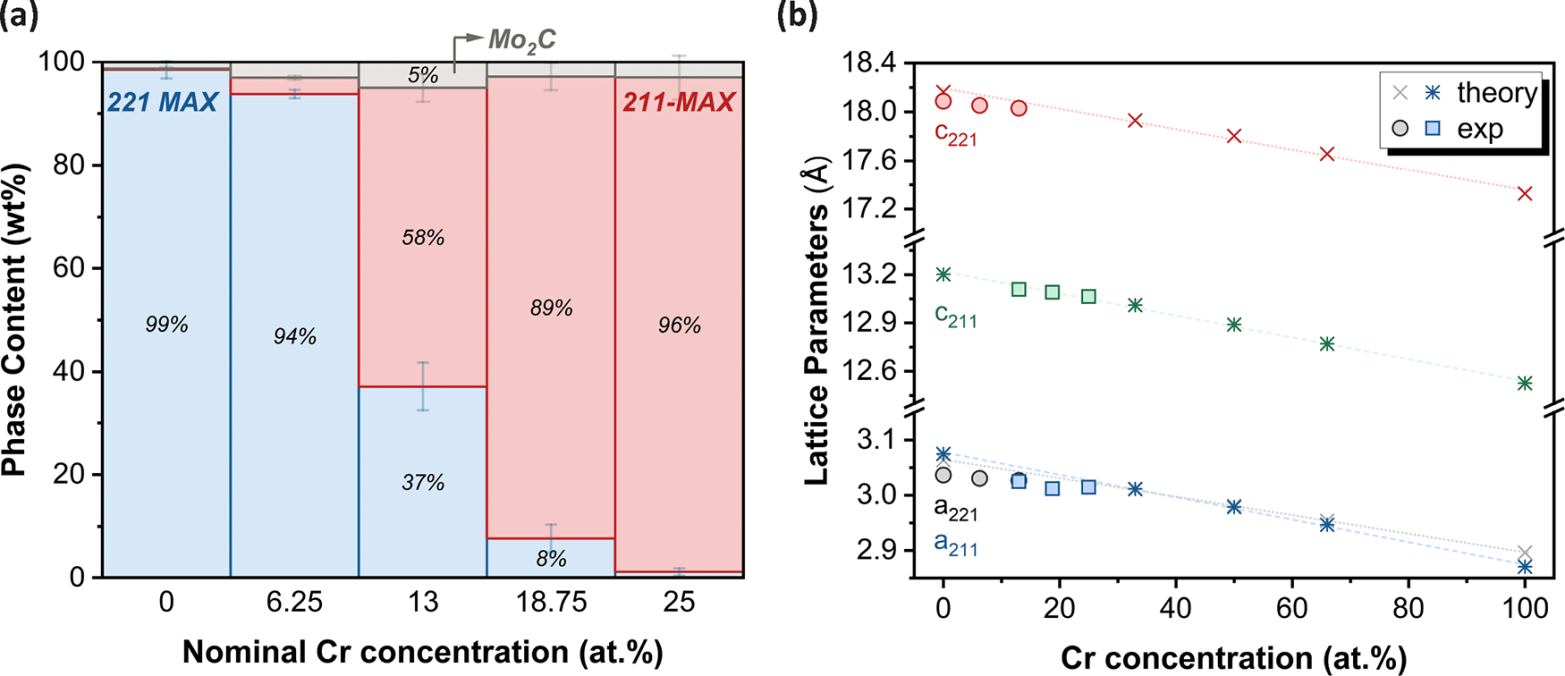
(a) Phase content of samples obtained through Rietveld analysis vs Cr content. (b) Summary of experimental and theoretically calculated lattice parameters for varying Cr concentration for 221 phases, i.e., Mo_2–*x*_Cr_*x*_Ga_2_C (circles and “×”, respectively) and 211 MAX-phases, i.e., Mo_2–*x*_Cr_*x*_GaC (squares and “*”, respectively).

A comparison of the lattice parameters for both MAX phases is shown in Figure 4b. All calculated lattice parameters agree with experimental results with less than 1% deviation. EDX measurements (Figure S8) also confirm the nominal Cr content, regardless of whether the 221 or 211 MAX phase is evaluated. Additional SEM analysis (Figure S9) reveals the typical layered morphology of the laminated MAX phase material, consistent with previous findings for the Mo_2_Ga_2_C system.^15,53^

Consistent with our previous findings,^15^ the metal ratios in both Mo_2–*x*_Cr_*x*_C and Mo_2–*x*_Cr_*x*_Ga_2_C MAX precursors are identical. This means that no Cr was lost to other impurity phases, which is a critical aspect for controlling defects in the subsequent MXene synthesis. Additionally, the linear relationship between the lattice parameters and the Cr content suggests that these atoms are randomly dispersed in the metal lattice. This observation was previously verified by TEM images on Mo_2–*x*_Cr_*x*_Ga_2_C (*x* = 0.26).^15^

### 3.4 MXenes Synthesis and Characterization

While both Mo_2_GaC and Mo_2_Ga_2_C phases are considered layered, only Mo_2_Ga_2_C has been shown to be exfoliable. Since the focus of the present study is defect engineering and controlled vacancy/pore formation in MXenes, only compounds consisting of predominantly the 221-MAX phase were chosen for the etching procedure. Consequently, the sample with 6.25 atom % Cr concentration was etched and compared with the 13 atom % Cr that had been produced previously, see ref 15. Samples with 19 and 25 atom % Cr had less than 10% 221-MAX phase content and were not considered for the etching step.

Figure 5a shows the XRD patterns of the 221-MAX phase and a free-standing film of Mo_1.87_CT_*z*_ MXene. It shows the typical increase of the interlayer spacing upon conversion of the 3D MAX phase into 2D MXene, as a result of the replacement of A-layer atoms with −O, −OH, and −F terminations and intercalated water molecules between the layers.^53^ The MAX to MXene conversion yield, calculated following ref 15 was 16 wt %, similar to previously reported 16 wt % for (Mo_2/3_Y_1/3_)_2_AlC *i*-MAX^54^ and Mo_1.87_Cr_0.13_Ga_2_C,^15^ and higher than previously shown 10 wt % for Mo_2_Ga_2_C.^53^

**Figure 5:**
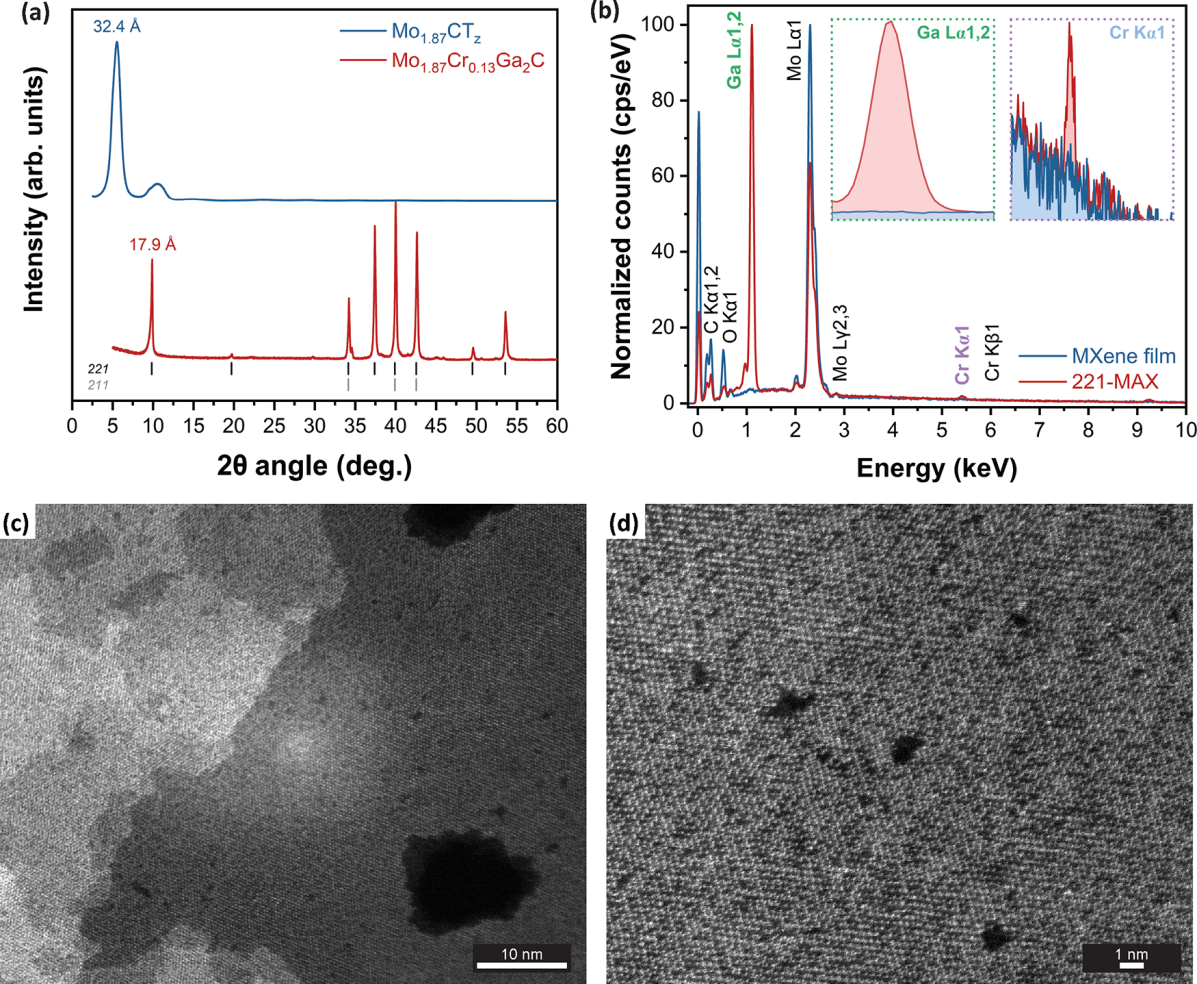
MXene Characterization. (a) XRD pattern for the 3D Mo_1.87_Cr_0.13_Ga_2_C powder sample and a filtered free-standing film of Mo_1.87_CT_*z*_ MXene (the small vertical lines below the patterns represent the peak positions for the 211 and 221-MAX phases). The values of the (002) peaks represent the double interlayer spacing (for the MAX phase corresponding to the c lattice parameter). (b) Representative EDX spectra for the 3D Mo_2–*x*_Cr_*x*_Ga_2_C powder particles and a filtered Mo_2–*x*_CT_*z*_ MXene film. (c) STEM image showing the range of pore distributions, from near single atom vacancies to pores of several nanometers. (d) Lattice resolved image to verify the small pores as well as the preservation of the hexagonal structure.

Figure 5b compares the EDX spectra of the 221-MAX phase alloyed with 6 atom % Cr and its corresponding MXene. Both the Ga and Cr peaks (highlighted in the insets) are no longer detectable in MXene, demonstrating their removal during the etching process. Quantitatively, the average Cr concentration decreases from 6.1 atom % in the 221-MAX phase to 0.3 atom % in the MXene film. Additionally, the Ga/Mo ratio in the MXene is zero (0.0) compared to a ratio of 1.0 in the 221-MAX phase. Thus, both Ga and Cr were removed during etching, consistent with previous reports for both ordered^55,56^ and disordered MAX phases,^14,15^ including previously synthesized Mo_1.74_CT_*z*_.^15^ Notably, in that case, X-ray photoelectron spectroscopy (XPS) evaluation also corroborated the complete removal of Ga and Cr. Furthermore, the XPS indicated oxygen as the predominant functional group and also showed that the Mo 3d and C 1s binding energies were very similar to the ones for Mo_4/3_CT_*z*_ *i*-MXene.^15^

The removal of Cr upon etching in acidic media has been demonstrated in both theoretical and experimental studies,^57^ showing that the Cr_2_AlC MAX phase is prone to dissolution of both Al and Cr, making it unlikely to form a MXene. Furthermore, our findings align with the removal of only the A-layer (not Mo) upon etching in other Mo-based MAX phases, such as Mo_2_Ga_2_C, Mo_2_TiAlC_2_, and Mo_2_Ti_2_AlC_3_.^58^

STEM was used to provide a more detailed view of the large (>1 μm) MXene single sheets obtained. A few holes with a diameter near 10 nm are present, but the majority of pores have a diameter around 2 nm, as can be seen in Figure 5c. These pores are smaller compared to those found in Nb_1.33_CT_*z*_ MXene (up to 4 nm size).^14^ Figure 5d shows a higher magnification from the same region, revealing that the structure of the MXene is preserved; however, a number of vacancies can be observed and the pores may be interpreted as clusters of vacancies.^59,60^ Compared with the MXene produced with 13 atom % Cr,^15^ the Mo_1.87_CT_*z*_ produced in the present paper displays a lower density of defects and a smaller pore size.

Based upon previous research,^61^ we hypothesize that the selective removal of Cr atoms may decrease the surrounding structural stability, making the material more prone to be dissolved in the acidic environment.^15^ Consequently, while the carbon and Mo contents likely remain unchanged for single-point vacancies (such as in Figure 5d), we hypothesize that both elements are expected to be removed in regions with pores or vacancy clusters (e.g., Figure 5c).

Selective etching is a complex chemical process encompassing several variables, and recent work has shown that the assessment of the chemical environment through the different chemical potentials is required to allow predictions of which MAX phases can be converted to MXene.^57,62^ While differences in bond strength and interlayer interactions cannot supply the same conclusive information, it may be used to provide deeper understanding of the etching process once experimentally verified. The bonding strength on the 221-phase was therefore evaluated by integrating the occupied states of the pCOHP curves (IpCOHP) for all interactions identified up to a cutoff distance of 4 Å (see Figures S4a and S4b). From the relative bond strength contribution, shown in Figure S4c, the M-C interaction is approximately 2.1 times as strong as that of M-Ga, regardless of Cr concentration. To compare Mo and Cr interactions avoiding the influence of the concentration of Cr and Mo in the solid solutions, the averaged IpCOHP was used (i.e., IpCOHP contribution of a certain bond divided by the number of interactions identified). As can be noted in Figure S4d, both Cr–Ga and Cr–C bonds are weaker than Mo–Ga and Mo–C, respectively, which is a result consistent with the selective removal of Cr together with Ga upon etching.

### 3.5 Electrochemical Characterization

CV, GCD, rate performance, cyclic stability, and EIS characterizations were conducted to further evaluate the defect-engineered Mo_1.87_CT_*z*_ MXene electrode as a potential candidate for electrochemical supercapacitor applications. The CV curves of Mo_1.87_CT_*z*_, with a potential window ranging from −0.5 to 0.3 V, display the typical pseudocapacitive behavior of MXenes, as evidenced by the distinct redox peaks (Figure 6a). The pair of redox peaks visible around −0.26 to 0.15 V originates from the pseudocapacitive characteristics of MXene through the redox activity of Mo, which explains the slow intrinsic electrochemical kinetics.^53,63^ The nearly symmetrical triangular GCD curves during charge and discharge, shown in Figure 6b for various current densities, highlight the electrode’s excellent capacitive performance and the rapid, reversible faradaic reactions of the Mo_2–*x*_CT_*z*_ MXene.

**Figure 6:**
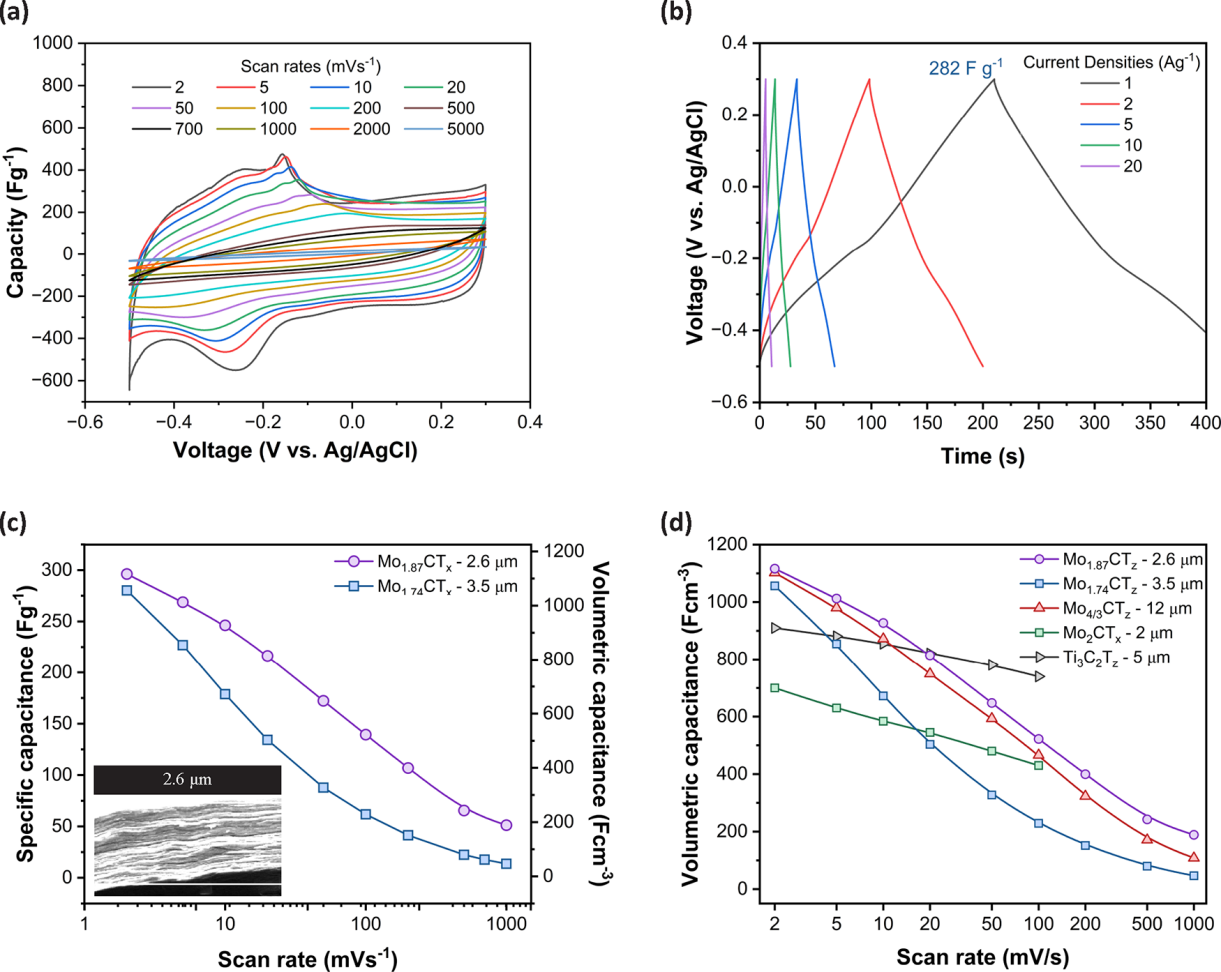
Electrochemical performance; (a) CV data for a 2.6 μm-thick Mo_2–*x*_CT_*z*_ MXene film, collected at scan rates from 2 to 5000 mV s^–1^; (b) GCD curves at different current density; (c) volumetric and gravimetric capacitance at different scan rates (the inset showing a cross section of a MXene film), and (d) comparison of the volumetric capacitance of the present work with previously reported values.^15,53,55,64^

At a scan rate of 2 mVs^–1^, the Mo_1.87_CT_*z*_ MXene achieves a maximum volumetric capacitance of 1117 Fcm^–3^ and a gravimetric capacitance of 304 Fg^1–^ (Figure 6c). The cyclic stability tests, depicted in Figure S10a, reveal that the Mo_1.87_CT_*z*_ MXene electrode exhibits excellent cyclic stability, retaining 71% of its capacitance after 10,000 charge/discharge cycles at a current density of 10 Ag^1–^. Notably, the Coulombic efficiency remains around 100% after 10,000 cycles, evidencing the electrode’s high electrochemical performance. Nyquist plots in Figure S10b show lower charge transfer resistance and ion transport resistance in the midhigh frequency region. Moreover, the nearly vertical orientation of the impedance imaginary part to the real part in the low-frequency region indicates ideal capacitive behavior.

The obtained volumetric capacitance is comparable to previously reported values for Mo_4/3_CT_*z*_ (1102 Fcm^–3^)^55^ and Mo_1.74_CT_*z*_ (1152 Fcm^–3^),^15^ and significantly higher than that of Mo_2_CT_*z*_ (700 Fcm^–3^)^53^ at the same scan rates, as shown in the comparison in Figure 6d. This suggests that the defects generated in Mo_2–*x*_CT_*z*_ or a change in surface chemistry resulting from defect formation,^65^ significantly enhance the capacitance—almost doubling the values reported for Mo_2_CT_*z*_. The cyclic stability of 71% (for *x* = 0.13) is slightly lower than the 79% obtained for a higher defect density (*x* = 0.26), which may be attributed to the lower number of pores in the MXene sheets. The pores likely help to alleviate stress during ion insertion and extraction, contributing to improved stability in materials with higher defect density.

Importantly, compounds with disordered vacancies/pores and those with ordered vacancies exhibit very similar capacitance curves, displaying excellent values at low scan rates, though with a noticeable decrease in capacitance at higher scan rates. This behavior contrasts with MXenes of ideal stoichiometry, such as Ti_3_C_2_T_*z*_ or Mo_2_CT_*z*_, which demonstrate better rate capability with a less pronounced decrease in capacitance at higher scan rates. Notably, a lower defect density in the Mo_1.87_CT_*z*_ MXene appears to improve the rate capability compared to the Mo_1.74_CT_*z*_ MXene.^15^

The electrochemical performance of our Mo_2_CT_*z*_ MXene is among the best reported for MXenes in acidic electrolytes, especially at low scan rates. Figure 6d includes Ti_3_C_2_T_*z*_ MXene as a benchmark, given its status as the most studied MXene and its reported high capacitance in acidic media.^64^ Other MXenes, such as Mo_2_TiC_2_T_*z*_, Mo_2_NT_*z*_, and V_4_C_3_T_*z*_, have demonstrated lower capacitances than Ti_3_C_2_T_*z*_ (and are therefore not shown in the graph). For a broader perspective on MXene electrochemical performance, readers are encouraged to consult refs 66,67 .

Altogether, we have demonstrated that using Cr as a sacrificial element in the Mo_2_Ga_2_C MAX phase is an effective strategy for defect engineering of MXenes. This approach not only advances the understanding of MXene synthesis and enlarges the property space, but may also provide valuable insights for the development and optimization of other defect-engineered 2D materials etched in acidic media, such as for the recently synthesized boridenes.^68,69^

Beyond Cr, it is anticipated that this method could be applicable to other elements, such as Sc, Zr, and Y, due to their removal during the etching process of other quaternary phases.^14,55,56^ This approach could also be extended to a variety of MAX phases, providing an additional parameter for controlling MXenes surface chemistry and properties. For instance, Sc has previously been shown to be removed from the (Nb_0.67_Sc_0.33_)_2_AlC phase, resulting in Nb_1.33_CT_*z*_ MXenes with randomly disordered vacancies.^14^

To identify promising systems, a more comprehensive understanding of the etching mechanisms of MXenes and sacrificial elements is essential. Insights could be gained using the new theoretical method described in refs 57,70 , which combines DFT calculations with tabulated formation energies of molecules and ions in aqueous solutions to predict selective etching of layered compounds in acidic media. Using this approach on MAX phases alloyed with potentially sacrificial elements could aid in defining MAX phases and MXenes suitable for defect engineering, as well as establish boundaries for defect generation. Kinetic studies may also improve the understanding of the synthesis of Mo_2–*x*_Cr_*x*_Ga_2_C, which may enable higher yield for increased Cr content.

The preliminary electrochemical results presented serve as a proof of concept of the importance of defects in the 2D material structure. Further studies are needed to fully elucidate the role of vacancies and pores in enhancing ion transport and accessibility. However, such evaluation is challenging due to introduced defects typically being accompanied by a change in surface terminations; i.e., a potential change in properties cannot be exclusively assigned to vacancies or pores alone. With this said, for further property evaluation connected to energy storage, using in situ characterization techniques could be employed to monitor material and structural changes within the electrode upon cycling, which together with DFT calculations could be used to provide insights into storage mechanisms and ion diffusion pathways.

Beyond the electrochemical properties shown here, vacancy-engineered MXenes may exhibit the potential for other applications. For instance, our recently discovered Mo_1.74_CT_*z*_ MXene demonstrated excellent results as cathode for rechargeable aqueous zinc-ion batteries, outperforming most of MXene-based aqueous zinc-ion batteries reported so far.^71^ Moreover, a controlled vacancy concentration could be beneficial for hydrogen evolution reactions (HER), as Mo_2_CT_*z*_ MXene has been identified as a promising catalyst^72,73^ and ordered vacancy *i*-MXenes have also shown encouraging HER activity.^11^

Despite the high potential of Mo-based MXenes, opportunities for improvement remain. Future research should focus on developing cost-effective and sustainable synthesis techniques that reduce excess Ga and shorten the lengthy synthesis process. Addressing these challenges will be crucial for potential upscaling, making Mo-based MXenes more attractive to industries looking for innovative solutions to modern technological challenges.

## 4 Conclusions

A simple and reproducible method for introducing random vacancies and pores into Mo-based MXenes has been further explored here through a combination of experiments and DFT calculations. Cr incorporation into 3D Mo_2–*x*_Cr_*x*_C was limited to approximately 60 atom % Cr, while subsequent synthesis of Mo_2–*x*_Cr_*x*_Ga_2_C required a lower Cr content (below 25 atom %) due to competing formation of the related Mo_2–*x*_Cr_*x*_GaC MAX phase. From 3D Mo_1.87_Cr_0.13_Ga_2_C, we synthesized 2D Mo_1.87_CT_*z*_ MXene with disordered vacancies and pores using an etching process that required shorter etching time while leading to improved yield compared to the stoichiometric compound. Initial electrochemical characterization demonstrated that defect formation facilitated volumetric and gravimetric capacitances of 1117 Fcm^–3^ and 304 Fg^1–^ at a scan rate of 2 mVs^–1^, respectively. These values surpass those of other MXenes without additives, including Mo_2_CT_*z*_. Furthermore, it is indicated that an optimized Cr content in the MAX phase with a resulting optimized defect generation in the MXene can influence the rate capability of the material. Lastly, we suggest that this method can be extended to other sacrificial elements and MAX phases, making MXene defect engineering a viable pathway for enhancing properties across various applications.

## Supplementary Material



## Notes

Additional computational details, including convergence tests, formation enthalpy details and COHP bond strength analyses; additional experimental details, including Rietveld refinements information, SEM images, and EDX measurements and electrochemical characterizations (PDF)
