# Supplementary material for: Defect Engineering of Mo2–x CT z MXenes through Precursor Alloying and Effects on Electrochemical Properties
Source: Chem Mater. 2025 May 30;37(11):4005–15. doi: 10.1021/acs.chemmater.5c00143 (PMC12159976; doi:10.1021/acs.chemmater.5c00143)
Supplement: Supplementary file 1 [file cm5c00143_si_001.pdf]

Supporting Information

Defect engineering of $Mo_{2-x}CT_z$ MXenes through precursor alloying and effects on electrochemical properties

Rodrigo M. Ronchi [1], Ningjun Chen [1], Joseph Halim,[1] Per O. Å. Persson [2], Johanna Rosen *[1]

[1] Materials Design division, Department of Physics, Chemistry, and Biology (IFM), Linköping University, SE-581 83 Linköping, Sweden.

[2] Thin Film Physics division, Department of Physics, Chemistry, and Biology (IFM), Linköping University, SE-581 83 Linköping, Sweden.

Corresponding authors: johanna.rosen@liu.se

CONTENT

Computational details **3**

Figure S 1: DFT convergence tests 3

Table S 1: DFT Comparison of ferromagnetic [F] and non-magnetic [NM] spin configurations ... 3

Figure S 2: Gibbs free energy of formation considering compositions close in phase space 4

Figure S 3: $Mo_{2-x}Cr_xC$ unitcell structures 4

Table S 2: Simulations of $Mo_{2-x}Cr_xC$ considering compositions close in phase space 5

Table S 3: Simulations of 221- and 211-MAX considering compositions close in phase space ... 6

Table S 4: Simulations of $Mo_{2-x}Cr_xC$ considering selected competing phases 7

Table S 5: Simulations of 221- and 211-MAX considering selected competing phases 8

Table S 6: Competing Phases included in the simulations. 9

Figure S 4: Bond strength analysis obtained by pCOHP for $Mo_{2-x}Cr_xGa_2C$ phases 12

Experimental Details I: Alloying Mo_2C with Cr **13**

Table S 7: Experimental synthesis powder ratios. 13

Figure S 5: $Mo_{2-x}Cr_xC$ XRD spectra with different Cr percentages. 13

Table S 8: Rietveld refinement summary for $Mo_{2-x}Cr_xC$. 14

Figure S 6: SEM images $Mo_{2-x}Cr_xC$ with different Cr percentages. 15

Experimental Details II: 221-MAX Phase **16**

Table S 9: Rietveld refinement summary of $Mo_{2-x}Cr_xGa_2C$, nominal Cr = 0%. 16

Table S 10: Rietveld refinement summary of $Mo_{2-x}Cr_xGa_2C$, nominal Cr = 6.25%. 17

Table S 11: Rietveld refinement summary of $Mo_{2-x}Cr_xGa_2C$, nominal Cr = 13%. 18

Table S 12: Rietveld refinement summary of $Mo_{2-x}Cr_xGa_2C$, nominal Cr = 18.75%. 19

Table S 13: Rietveld refinement summary of $Mo_{2-x}Cr_xGa_2C$, nominal Cr = 25%. 20

Figure S 7: Phase contents for different synthesis temperatures 21

Figure S 8: EDX Measurements of Cr at% for both 211 and 221-MAX phases 21

Figure S 9: SEM images of 221-MAX Phases with different Cr percentages. 22

Experimental Details III: $Mo_{2-x}CT_z$ MXenes **23**

Figure S 10. Electrochemical characterization of a vacuum-filtered $Mo_{1.87}CT_z$ film. 23

Computational details

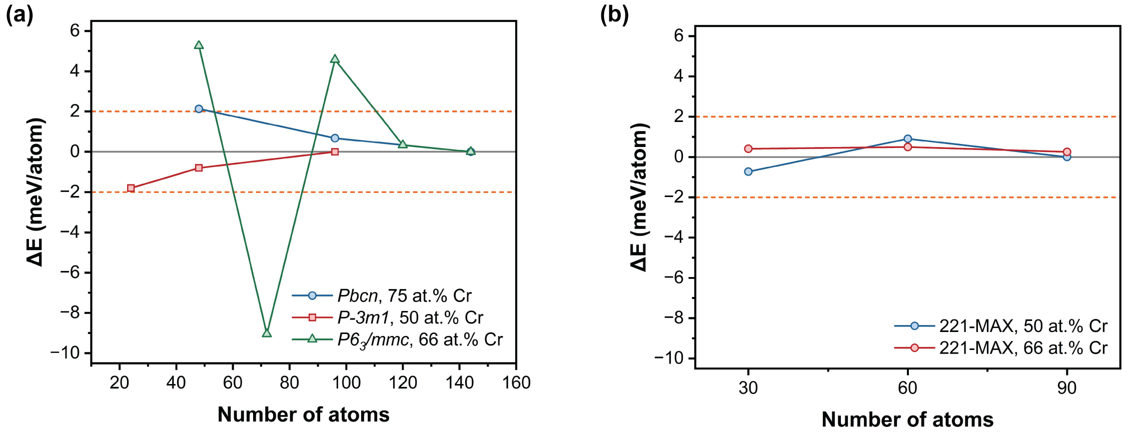

Figure S 1: DFT convergence tests: Difference of DFT calculated energies with respect to the largest supercell size ('ΔE') for (a) $Mo_{2-x}Cr_xC$ (one Cr content for three different space groups) and (b) $Mo_{2-x}Cr_xGa_2C$ (two different Cr compositions with one space group only). Dashed lines represent the ±2 meV/atom convergence criteria used.

Table S 1: Comparison of ferromagnetic [F] and non-magnetic [NM] spin configurations evaluated by DFT. The magnetization of the ferromagnetic configuration is provided to show that the system converges to the NM configuration.

**Phase**	**Notation**	**E [F] – E [NM] (meV/atom)**	**Mag per atom [F] (μ_B/atom)**
Mo_2C[*Pbcn*]	Precursor	0.0	-0.00
Cr_2C[*Pbcn*]	Precursor	0.0	0.00
Mo_2GaC	211	0.0	0.00
Cr_2GaC	211	0.0	-0.00
Mo_2Ga_2C	221	0.0	-0.00
$Mo_{0.33}Cr_{0.67}Ga_2C$	221	0.0	0.01
Cr_2Ga_2C	221	0.0	-0.00

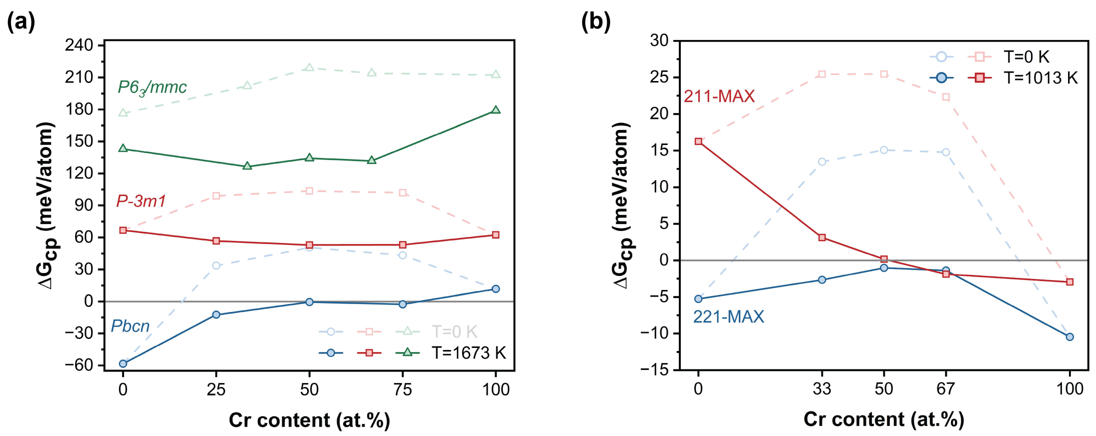

Figure S 2: Calculated Gibbs free energy of formation, ΔG_{cp}, considering competing phases with compositions close in phase space. (a) $Mo_{2-x}Cr_xC$ and (b) $Mo_{2-x}Cr_xGa_2C$ (221) and $Mo_{2-x}Cr_xGaC$ (211) MAX phases.

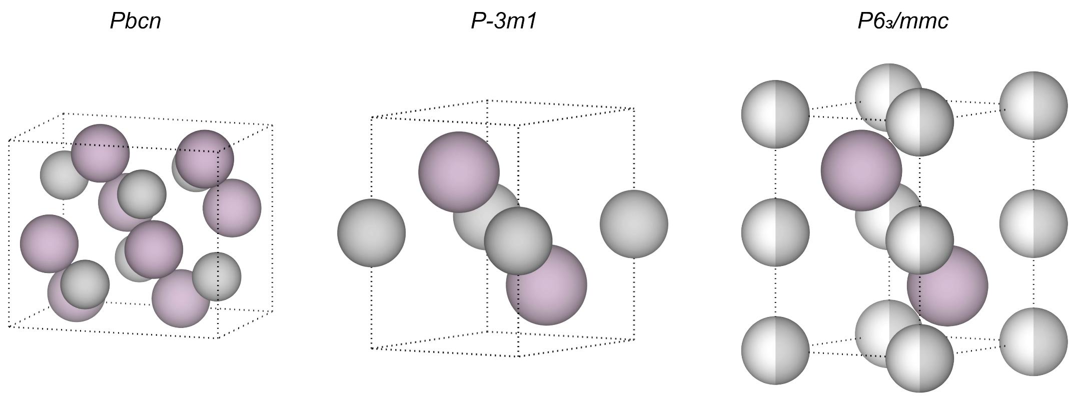

Figure S 3: $Mo_{2-x}Cr_xC$ unit cell structures for the three space groups evaluated.

Table S 2: $Mo_{2-x}Cr_xC$ simulations considering competing phases with compositions close in phase space. DFT energies (0 K), Gibbs formation enthalpy (ΔG_{cp}) at T=1673K and list of competing phases for each space group and Cr concentration.

Name	Cr Conc	SG	E_{DFT} (eV/atom)	ΔG_{cp} (meV/atom)	$minE_{cp}$ (eV/atom)	List of Competing phases
Mo_2C	0	*P-3m1*	-10.353	67	-10.420	Mo_2C [*Pbcn*]
Mo_2C	0	*P6₃/mmc*	-10.244	143	-10.420	Mo_2C [*Pbcn*]
Mo_2C	0	*Pbcn*	-10.420	-58	-10.362	MoC [*P-6m2*] + Mo [*Im-3m*]
$Mo_{1.5}Cr_{0.5}C$	1/4	*Pbcn*	-10.184	-12	-10.226	Mo_2C [*Im-3m*] + $(Mo_{0.5}Cr_{0.5})_2C$ [*Pbcn*]
$Mo_{1.33}Cr_{0.67}C$	1/3	*P-3m1*	-10.051	57	-10.169	$Mo_{0.5}Cr_{0.5}C$ [*Pbcn*] + $Cr_{0.25}Mo_{0.75}C_2$ [*Pbcn*]
$Mo_{1.33}Cr_{0.67}C$	1/3	*P6₃/mmc*	-9.948	126	-10.169	$Mo_{0.5}Cr_{0.5}C$ [*Pbcn*] + $Cr_{0.25}Mo_{0.75}C_2$ [*Pbcn*]
$Mo_{0.5}Cr_{0.5}C$	1/2	*p-3m1*	-9.912	53	-10.032	$Mo_{0.5}Cr_{0.5}C$ [*Pbcn*]
$Mo_{0.5}Cr_{0.5}C$	1/2	*P6₃/mmc*	-9.797	135	-10.031	$Mo_{0.5}Cr_{0.5}C$ [*Pbcn*]
$Mo_{0.5}Cr_{0.5}C$	1/2	*Pbcn*	-9.965	0	-10.031	$Cr_{0.75}Mo_{0.25}C_2$ [*Pbcn*] + $Cr_{0.25}Mo_{0.75}C_2$ [*Pbcn*]
$Mo_{0.67}Cr_{1.33}C$	2/3	*P-3m1*	-9.779	53	-9.893	$Mo_{0.5}Cr_{0.5}C$ [*Pbcn*] + Cr_3MoC_2 [*Pbcn*]
$Mo_{0.67}Cr_{1.33}C$	2/3	*P6₃/mmc*	-9.667	132	-9.893	$Mo_{0.5}Cr_{0.5}C$ [*Pbcn*] + Cr_3MoC_2 [*Pbcn*]
$Mo_{0.5}Cr_{1.5}C_2$	3/4	*Pbcn*	-9.770	-3	-9.821	$Mo_{0.5}Cr_{0.5}C$ [*Pbcn*] + Cr_7C_3 [*P6₃mc*] + Cr_3C_2 [*Pnma*]
Cr_2C	1	*P-3m1*	-9.549	62	-9.611	Cr_3C_2 [*Pnma*] + Cr_7C_3 [*P6₃mc*]
Cr_2C	1	*P6₃/mmc*	-9.399	179	-9.611	Cr_3C_2 [*Pnma*] + Cr_7C_3 [*P6₃mc*]
Cr_2C	1	*Pbcn*	-9.599	12	-9.611	Cr_3C_2 [*Pnma*] + Cr_7C_3 [*P6₃mc*]

Table S 3: 221- and 211-MAX simulations considering competing phases with compositions close in phase space. DFT energies (0 K), Gibbs formation enthalpy (ΔG_{cp}) at T=1013K and list of competing phases for each phase and Cr concentration. These two phases have the $P6_3/mmc$ space group.

Name	Phase	Conc	E_{DFT} (eV/atom)	ΔG_{cp} (meV/atom)	$minE_{cp}$ (eV/atom)	List of Competing phases
Mo_2GaC	211	0	-8.603	16	-8.619	Mo_2Ga_2C [*P6₃/mmc*] + Mo_2C [*Pbcn*]
$(Mo_{0.67}Cr_{0.33})_2GaC$	211	1/3	-8.396	3	-8.426	$(Mo_{0.5}Cr_{0.5})_2Ga_2C$ [$P6_3/mmc$] + $(Mo_{0.33}Cr_{0.67})_2GaC$ [$P6_3/mmc$] + Mo_2C [*Pm-3n*]
$Mo_{0.5}Cr_{0.5}GaC$	211	1/2	-8.296	0	-8.327	$(Mo_{0.5}Cr_{0.5})_2Ga_2C$ [$P6_3/mmc$] + $(Mo_{0.33}Cr_{0.67})_2GaC$ [$P6_3/mmc$] + Mo_2C [*Pm-3n*]
$(Mo_{0.33}Cr_{0.67})_2GaC$	211	2/3	-8.199	-2	-8.225	Cr_3C_2 [*Pnma*] + $Mo_{0.5}Cr_{0.5}GaC$ [$P6_3/mmc$] + $(Mo_{0.33}Cr_{0.67})_2Ga_2C$ [$P6_3/mmc$] + $GaMo_3$ [*Pm-3n*]
Cr_2GaC	211	1	-8.021	-3	-8.018	Cr_7C_3 [$P6_3mc$] + Cr_3C_2 [*Pnma*] + Cr_2Ga_2C [$P6_3/mmc$]
Mo_2Ga_2C	221	0	-7.539	-5	-7.534	Mo_2C [*Pbcn*] + C [R-3m] + Ga_4Mo [*Im-3m*]
$(Mo_{0.67}Cr_{0.33})_2Ga_2C$	221	1/3	-7.367	-3	-7.386	$(Mo_{0.5}Cr_{0.5})_2Ga_2C$ [$P6_3/mmc$] + Mo_2Ga_2C [$P6_3/mmc$]
$(Mo_{0.5}Cr_{0.5})_2Ga_2C$	221	1/2	-7.286	-1	-7.309	$(Mo_{0.67}Cr_{0.33})_2Ga_2C$ [$P6_3/mmc$] + $(Mo_{0.33}Cr_{0.67})_2Ga_2C$ [$P6_3/mmc$]
$(Mo_{0.33}Cr_{0.67})_2Ga_2C$	221	2/3	-7.207	-1	-7.228	Cr_2Ga_2C [$P6_3/mmc$] + $(Mo_{0.5}Cr_{0.5})_2Ga_2C$ [$P6_3/mmc$]
Cr_2Ga_2C	221	1	-7.063	-10	-7.052	$CrGa_4$ [*Im-3m*] + 1/14 [*R-3m*] + Cr_2GaC [$P6_3/mmc$]

Table S 4: $Mo_{2-x}Cr_xC$ simulations considering selected competing phases. DFT energies (0 K), Gibbs formation enthalpy (ΔG_{cp}) at T=1673K and list of competing phases for each space group and Cr concentration.

Name	Conc	SG	E_{DFT} (eV/atom)	ΔG_{cp} (meV/atom)	$minE_{cp}$ (eV/atom)	CPs info
Mo_2C	0	*P-3m1*	-10.353	67	-10.420	Mo_2C [*Pbcn*]
Mo_2C	0	*P6₃/mmc*	-10.244	143	-10.420	Mo_2C [*Pbcn*]
Mo_2C	0	*Pbcn*	-10.420	-58	-10.362	MoC [*P-6m2*] + Mo [*Im-3m*]
$Mo_{1.5}Cr_{0.5}C$	1/4	*Pbcn*	-10.184	-20	-10.218	Cr_7C_3 [$P6_3mc$] + Cr_3C_2 [*Pnma*] + Mo_2C [*Pbcn*]
$Mo_{1.33}Cr_{0.67}C$	1/3	*P-3m1*	-10.051	38	-10.150	Cr_7C_3 [$P6_3mc$] + Cr_3C_2 [*Pnma*] + Mo_2C [*Pbcn*]
$Mo_{1.33}Cr_{0.67}C$	1/3	$P6_3/mmc$	-9.948	108	-10.150	Cr_7C_3 [$P6_3mc$] + Cr_3C_2 [*Pnma*] + Mo_2C [*Pbcn*]
$Mo_{0.5}Cr_{0.5}C$	1/2	*p-3m1*	-9.912	37	-10.016	Cr_7C_3 [$P6_3mc$] + Cr_3C_2 [*Pnma*] + Mo_2C [*Pbcn*]
$Mo_{0.5}Cr_{0.5}C$	1/2	$P6_3/mmc$	-9.797	119	-10.016	Cr_7C_3 [$P6_3mc$] + Cr_3C_2 [*Pnma*] + Mo_2C [*Pbcn*]
$Mo_{0.5}Cr_{0.5}C$	1/2	*Pbcn*	-9.965	-16	-10.016	Cr_7C_3 [$P6_3mc$] + Cr_3C_2 [*Pnma*] + Mo_2C [*Pbcn*]
$Mo_{0.67}Cr_{1.33}C$	2/3	*P-3m1*	-9.779	40	-9.881	Cr_7C_3 [$P6_3mc$] + Cr_3C_2 [*Pnma*] + Mo_2C [*Pbcn*]
$Mo_{0.67}Cr_{1.33}C$	2/3	$P6_3/mmc$	-9.667	119	-9.881	Cr_7C_3 [$P6_3mc$] + Cr_3C_2 [*Pnma*] + Mo_2C [*Pbcn*]
$Mo_{0.5}Cr_{1.5}C_2$	3/4	*Pbcn*	-9.770	-11	-9.813	Cr_7C_3 [$P6_3mc$] + Cr_3C_2 [*Pnma*] + Mo_2C [*Pbcn*]
Cr_2C	1	*P-3m1*	-9.549	62	-9.611	Cr_3C_2 [*Pnma*] + Cr_7C_3 [$P6_3mc$]
Cr_2C	1	$P6_3/mmc$	-9.399	179	-9.611	Cr_3C_2 [*Pnma*] + Cr_7C_3 [$P6_3mc$]
Cr_2C	1	*Pbcn*	-9.599	12	-9.611	Cr_3C_2 [*Pnma*] + Cr_7C_3 [$P6_3mc$]

Table S 5: 221- and 211-MAX simulations considering selected competing phases. DFT energies (0 K), Gibbs formation enthalpy (ΔG_{cp}) at T=1013K and competing phases information for each phase and Cr concentration. These two phases have $P6_3/mmc$ space group.

Name	Phase	Conc	E_{DFT} (eV/atom)	ΔG_{cp} (meV/atom)	$minE_{cp}$ (eV/atom)	CPs info
Mo_2GaC	211	0	-8.603	16	-8.619	Mo_2Ga_2C [P63/mmc] + Mo_2C [*Pbcn*]
$(Mo_{0.67}Cr_{0.33})_2GaC$	211	1/3	-8.396	2	-8.425	Cr_3C_2 [*Pnma*] + $(Mo_{0.67}Cr_{0.33})_2Ga_2C$ [P63/mmc] + $GaMo_3$ [*Pm-3n*] + Mo_2C [*Pbcn*]
$Mo_{0.5}Cr_{0.5}GaC$	211	1/2	-8.296	-1	-8.326	Cr_3C_2 [*Pnma*] + $(Mo_{0.5}Cr_{0.5})_2Ga_2C$ [P63/mmc] + $GaMo_3$ [*Pm-3n*] + Mo_2C [*Pbcn*]
$(Mo_{0.33}Cr_{0.67})_2GaC$	211	2/3	-8.199	-2	-8.225	Cr_3C_2 [*Pnma*] + $(Mo_{0.67}Cr_{0.33})_2Ga_2C$ [P63/mmc] + $GaMo_3$ [*Pm-3n*] + Mo_2C [*Pbcn*]
Cr_2GaC	211	1	-8.021	-3	-8.018	Cr_7C_3 [P6_3mc] + Cr_3C_2 [*Pnma*] + Cr_2Ga_2C [P63/mmc]
Mo_2Ga_2C	221	0	-7.539	-5	-7.534	Mo_2C [*Pbcn*] + C [*R-3m*] + Ga4Mo [*Im-3m*]
$(Mo_{0.67}Cr_{0.33})_2Ga_2C$	221	1/3	-7.367	-9	-7.380	Mo_2Ga_2C [P63/mmc] + Cr_2Ga_2C [P63/mmc]
$(Mo_{0.5}Cr_{0.5})_2Ga_2C$	221	1/2	-7.286	-9	-7.301	$Mo_{0.5}Cr_{0.5}GaC$ [P63/mmc] + Cr_2Ga_2C [P63/mmc] + C [*R-3m*] + Ga_4Mo [*Im-3m*]
$(Mo_{0.33}Cr_{0.67})_2Ga_2C$	221	2/3	-7.207	-7	-7.222	Cr_2Ga_2C [P63/mmc] $(Mo_{0.33}Cr_{0.67})_2GaC$ [P63/mmc] + C [*R-3m*] + Ga_4Mo [*Im-3m*]
Cr_2Ga_2C	221	1	-7.063	-10	-7.052	$CrGa_4$ [*Im-3m*] + C [*R-3m*] + Cr_2Ga_2C [P63/mmc]

Table S 6: Competing Phases included in the simulations

	Name	E(eV)	Space Group	ID	E(eV/atom)
0	C12	-109.010	*P6_3/mmc*	mp-611448	-9.084
1	C2	-13.242	*Cmcm*	mp-1097832	-6.621
2	C120	-1023.524	*Pnnm*	mp-1205283	-8.529
3	C8	-71.687	*Cmmm*	mp-1078845	-8.961
4	C8	-67.150	*Ia-3*	mp-24	-8.394
5	C100	-823.297	*P1*	mp-1244913	-8.233
6	C4	-35.161	*Cmmm*	mp-1008374	-8.790
7	C8	-66.269	*Cmmm*	mp-579909	-8.284
8	C	-6.592	*I4/mmm*	mp-1181996	-6.592
9	C28	-216.280	*P2_1*	mp-1194362	-7.724
10	C20	-169.269	*Pm-3m*	mp-1188817	-8.463
11	C80	-615.920	*P2_12_12_1*	mp-1182684	-7.699
12	C80	-625.453	*P1*	mp-1197903	-7.818
13	C4	-36.882	*Cmme*	mp-568286	-9.220
14	C140	-1242.955	*Cmcm*	mp-683919	-8.878
15	C4	-35.574	*I4/mmm*	mp-1008395	-8.894
16	C8	-73.584	*R-3m*	mp-569416	-9.198
17	C2	-18.433	*P6/mmm*	mp-568806	-9.217
18	C12	-110.621	*P6_3/mmc*	mp-606949	-9.218
19	C8	-72.648	*P6_3/mmc*	mp-611426	-9.081
20	C120	-1060.797	*Pnnm*	mp-1147718	-8.840
21	C8	-67.705	*Im-3m*	mp-570002	-8.463
22	C4	-31.685	*I4_132*	mp-1018088	-7.921
23	C16	-145.370	*P6_3/mmc*	mp-616440	-9.086
24	C2	-18.438	*P6/mmm*	mp-1040425	-9.219
25	C2	-18.445	*Fmmm*	mp-937760	-9.223
26	C2	-18.438	*P6/mmm*	mp-1040425	-9.219
27	C71	-646.866	*Cm*	mp-1096869	-9.111
28	C120	-1043.736	*Pnnm*	mp-568028	-8.698
29	C2	-18.181	*Fd-3m*	mp-66	-9.090
30	C4	-36.876	*P6_3/mmc*	mp-997182	-9.219
31	C8	-71.422	*C2/m*	mp-1080826	-8.928
32	C48	-392.848	*P2_1/c*	mp-1203645	-8.184
33	C	-8.226	*P2/m*	mp-1182029	-8.226
34	C	-6.464	*Pm-3m*	mp-998866	-6.464
35	C4	-36.261	*P6_3/mmc*	mp-47	-9.065
36	C60	-529.986	*Immm*	mp-630227	-8.833
37	C20	-161.201	*I4/mmm*	mp-1205417	-8.060
38	C100	-829.273	*P1*	mp-1244964	-8.293
39	C10	-90.821	*R-3m*	mp-569517	-9.082
40	C12	-97.264	*I4/mmm*	mp-1095534	-8.105
41	C240	-2122.471	*Pa-3*	mp-1196583	-8.844
42	C60	-529.171	*R-3m*	mp-680372	-8.820
43	C2	-18.451	*R-3m*	mp-169	-9.225

44	C100	-821.359	*P1*	mp-1245190	-8.214
45	C8	-63.781	*Cmme*	mp-624889	-7.973
46	C60	-530.279	*Fm-3*	mp-667273	-8.838
47	C4	-36.882	*Cmme*	mp-568286	-9.220
48	C2	-13.190	*Cmcm*	mp-1056957	-6.595
49	C4	-36.907	*R-3m*	mp-569304	-9.227
50	C8	-69.758	*Cmmm*	mp-568410	-8.720
51	C14	-127.154	*R-3m*	mp-569567	-9.082
52	C4	-36.881	*P6_3/mmc*	mp-48	-9.220
53	C12	-98.759	*I4/mmm*	mp-1095633	-8.230
54	C2	-18.429	*C2/m*	mp-632329	-9.215
55	C4	-36.876	*P6_3/mmc*	mp-997182	-9.219
56	C16	-142.976	*Pnma*	mp-1190171	-8.936
57	C29	-211.943	*I-43m*	mp-1192619	-7.308
58	Cr	-9.249	*Fm-3m*	mp-8633	-9.249
59	Cr8	-76.542	*Pm-3n*	mp-17	-9.568
60	Cr2	-18.628	*Cmcm*	mp-1059289	-9.314
61	Cr2	-19.306	*Im-3m*	mp-90	-9.653
62	Cr2	-18.465	*P6_3/mmc*	mp-89	-9.233
63	Cr56C24	-770.001	*P6_3mc*	mp-1196316	-9.625
64	Cr28C12	-384.977	*Pnma*	mp-19855	-9.624
65	Cr12C4	-153.893	*Pnma*	mp-1189286	-9.618
66	CrC	-18.491	*Fm-3m*	mp-579	-9.245
67	Cr23C6	-279.458	*Fm-3m*	mp-723	-9.636
68	Cr12C8	-191.656	*Pnma*	mp-20937	-9.583
69	Cr2C	-28.645	*P-3m1*	mp-1226378	-9.548
70	Cr6C4	-95.524	*Cmcm*	mp-570112	-9.552
71	CrC	-18.888	*P-6m2*	mp-1018050	-9.444
72	Cr2C	-28.646	*P-3m1*	this work	-9.549
73	Mo	-10.419	*Fm-3m*	mp-8637	-10.419
74	Mo	-9.975	*P6/mmm*	mp-1056004	-9.975
75	Mo	-10.846	*Im-3m*	mp-129	-10.846
76	Mo4	-41.614	*P6_3/mmc*	mp-1066523	-10.404
77	MoC	-19.074	*F-43m*	mp-1009218	-9.537
78	Mo4C3	-70.386	*R-3m*	mp-1221488	-10.055
79	Mo6C4	-101.374	*P-3m1*	mp-1221489	-10.137
80	Mo6C6	-118.399	*P6_3/mmc*	mp-15798	-9.867
81	Mo4C4	-79.255	*P6_3/mmc*	mp-567925	-9.907
82	Mo3C2	-50.833	*P-3m1*	mp-1221473	-10.167
83	Mo2C	-29.284	*P-6m2*	mp-571589	-9.761
84	Mo2C	-31.058	*P-3m1*	mp-1221498	-10.353
85	MoC	-19.646	*Fm-3m*	mp-2746	-9.823
86	MoC	-20.240	*P-6m2*	mp-2305	-10.120
87	Mo2C	-31.060	*P-3m1*	this work	-10.353
88	Cr8C4	-115.191	*Pbcn*	this work	-9.599
89	Mo8C4	-125.030	*Pbcn*	mp-1552	-10.419

90	Mo8C4	-125.042	*Pbcn*	this work	-10.420
91	Ga4	-12.051	*Cmce*	mp-1007857	-3.013
92	Ga20	-60.328	*Cmcm*	mp-567540	-3.016
93	Ga4	-12.093	*Cmcm*	mp-1067880	-3.023
94	Ga6	-18.072	*I-43d*	mp-569423	-3.012
95	Ga2	-6.022	*Cmcm*	mp-10021	-3.011
96	Ga4	-12.112	*Cmce*	mp-142	-3.028
97	Ga22	-65.869	*R-3m*	mp-569007	-2.994
98	Ga	-2.999	*I4/mmm*	mp-140	-2.999
99	Ga2C2	-20.528	*P6_3mc*	mp-1184015	-5.132
100	GaC3	-17.820	*Pm-3m*	mp-1064861	-4.455
101	Cr6Ga2	-64.309	*Pm-3n*	mp-1231	-8.039
102	Cr13Ga13	-165.257	*R-3m*	mp-1182452	-6.356
103	Cr9Ga12	-123.734	*C2/m*	mp-18654	-5.892
104	CrGa4	-22.547	*Im-3m*	mp-1070544	-4.509
105	Cr4Ga2C2	-64.167	*P6_3/mmc*	mp-20197	-8.021
106	Cr4Ga2C2	-64.171	*P63/mmc*	this work	-8.021
107	Ga2Mo6	-72.453	*Pm-3n*	mp-2377	-9.057
108	Ga62Mo12	-327.947	*P-1*	mp-1203833	-4.432
109	Ga41Mo8	-217.450	*R-3*	mp-1195805	-4.438
110	Ga4Mo	-23.832	*Im-3m*	this work	-4.766
111	Ga40Mo8C	-220.491	*R-3*	mp-1196543	-4.500
112	Ga2Mo4C2	-68.819	*P6_3/mmc*	mp-1079635	-8.602
113	Ga2Mo4C2	-68.825	*P63/mmc*	this work	-8.603
114	Cr4Ga4C2	-70.628	*P63/mmc*	this work	-7.063
115	Ga4Mo4C2	-75.389	*P63/mmc*	this work	-7.539
116	Cr3Mo2C4	-86.386	*R3m*	mp-1226369	-9.598
117	Cr22MoC6	-279.853	*Fm-3m*	mp-1193982	-9.650
118	Cr21Mo2C6	-282.285	*Fm-3m*	mp-641573	-9.734
119	Cr32Mo32C32	-956.631	*Pbcn*	this work	-9.965
120	Cr56Mo8C32	-929.289	*Pbcn*	this work	-9.680
121	Cr48Mo16C32	-937.905	*Pbcn*	this work	-9.770
122	Cr16Mo48C32	-977.682	*Pbcn*	this work	-10.184
123	Cr24Mo48C36	-1085.549	*P-3m1*	this work	-10.051
124	Cr32Mo32C32	-951.547	*P-3m1*	this work	-9.912
125	Cr48Mo24C36	-1056.108	*P-3m1*	this work	-9.779
126	Cr20Mo40C30	-895.355	*P63/mmc*	this work	-9.948
127	Cr32Mo32C32	-940.494	*P63/mmc*	this work	-9.797
128	Cr40Mo20C30	-870.006	*P63/mmc*	this work	-9.667
129	Cr24Ga36Mo12C18	-648.609	*P63/mmc*	this work	-7.207
130	Cr18Ga36Mo18C18	-655.722	*P63/mmc*	this work	-7.286
131	Cr16Ga24Mo32C24	-805.977	*P63/mmc*	this work	-8.396
132	Cr32Ga24Mo16C24	-787.137	*P63/mmc*	this work	-8.199
133	Cr24Ga24Mo24C24	-796.453	*P63/mmc*	this work	-8.296
134	Cr12Ga36Mo24C18	-663.008	*P63/mmc*	this work	-7.367

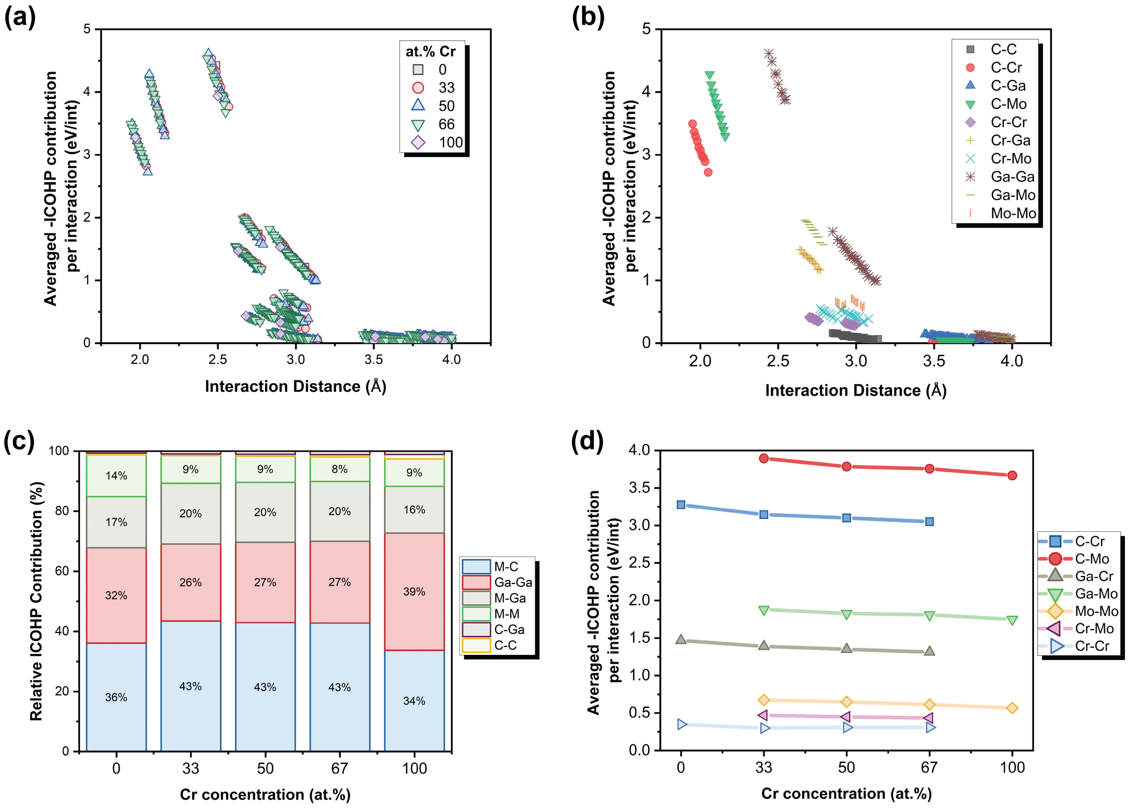

Figure S 4: Bond strength analysis obtained by pCOHP for $Mo_{2-x}Cr_xGa_2C$ phases. (a, b) Averaged –IpCOHP contributions as a function of the interaction distance on $Mo_{2-x}Cr_xGa_2C$ phases for different Cr content (a) and specific 50 at.% Cr content, showing the different bonds identified (b). Note that the contribution becomes almost negligible at around 3.5 Å. (c) IpCOHP contribution, in percentage, up to the Fermi energy for all interactions with respect to the total net bonding. (d) Averaged –IpCOHP contributions for selected bonds. Note that all Cr bonds have lower contribution compared to the respective Mo ones.

Experimental Details I: Alloying Mo_2C with Cr

Table S 7: Experimental synthesis powder atomic ratios. Cr concentration column refers to the metal site.

Cr concentration (at.% Cr)	Mo	Cr	C
0	2	0	1
6.25	1.875	0.125	1
13	1.74	0.26	1
18.75	1.625	0.375	1
25	1.5	0.5	1
33	1.33	0.67	1
50	1	1	1
66.6	0.67	1.33	1
87.5	0.25	1.75	1

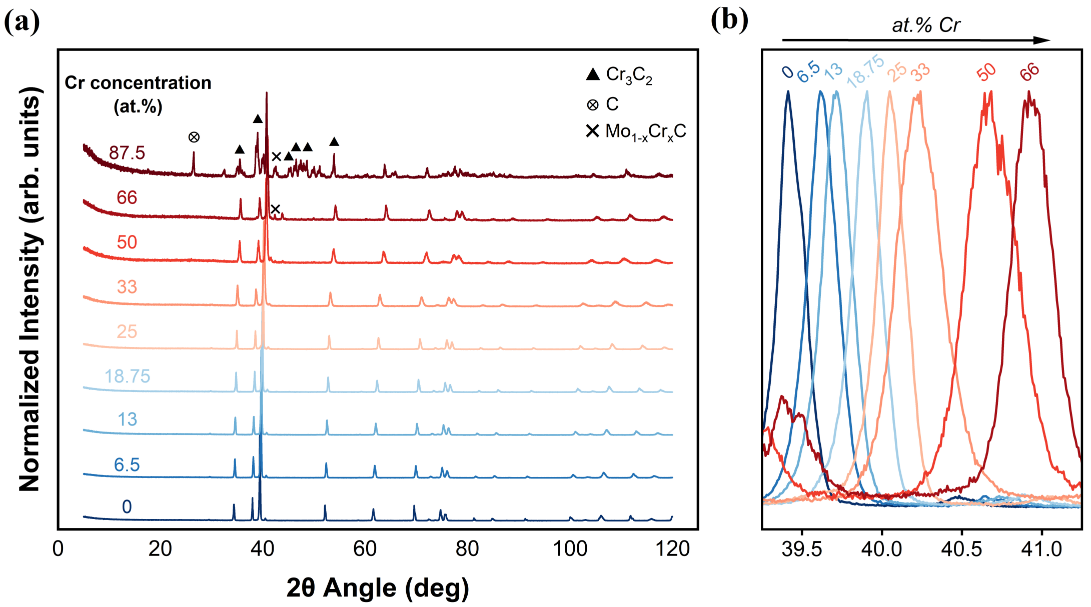

Figure S 5: $Mo_{2-x}Cr_xC$ XRD spectra with different Cr percentages. (a) Only main impurities peaks are labelled to make the image clearer (Mo_2C labels are not shown since from 0 to 50 Cr% samples are phase pure). (b) Region around 40 degrees showing the peak shift to larger angles with increasing Cr content (87.5Cr% is not shown).

Table S 8: Rietveld refinement summary for $Mo_{2-x}Cr_xC$. The value in parentheses is the error (if not present, the parameter was not refined). Only the Mo_2C phase is shown since this is the majority phase. The main impurity phase identified was $Mo_{1-x}Cr_x$ solid solution.

Cr conc (at.%)	0	6.25	13	18.75	25	33	50	66
Space group	*Pbcn* (#60)	*Pbcn* (#60)	*Pbcn* (#60)	*Pbcn* (#60)	*Pbcn* (#60)	*Pbcn* (#60)	*Pbcn* (#60)	*Pbcn* (#60)
a (Å)	4.73212(2)	4.71966(3)	4.70176(3)	4.68553(7)	4.67271(3)	4.6578(1)	4.6034(1)	4.5676(1)
b (Å)	6.02681(3)	6.00864(4)	5.98448(5)	5.96368(9)	5.94342(5)	5.9366(2)	5.8240(2)	5.8168(2)
c (Å)	5.20496(3)	5.18940(4)	5.16726(4)	5.14989(8)	5.13547(4)	5.1085(1)	5.0774(1)	5.0180(1)
α	90.00	90.00	90.00	90.00	90.00	90.00	90.00	90.00
β	90.00	90.00	90.00	90.00	90.00	90.00	90.00	90.00
γ	90.00	90.00	90.00	90.00	90.00	90.00	90.00	90.00
Mo (8d)	Occ = 8.00<br>0.2437(4)<br>0.1281(3)<br>0.0816(2)	Occ = 7.50<br>0.2452(4)<br>0.1278(4)<br>0.0820(3)	Occ = 6.96<br>0.2444(4)<br>0.1290(3)<br>0.0821(3)	Occ = 6.50<br>0.2471(6)<br>0.1286(4)<br>0.0822(3)	Occ = 6.00<br>0.2454(5)<br>0.1280(4)<br>0.0822(3)	Occ = 5.33<br>0.2437(2)<br>0.1281(3)<br>0.0816(2)	Occ = 4.00<br>0.2549(7)<br>0.1287(7)<br>0.0835(4)	Occ = 2.67<br>0.2430(7)<br>0.1270(15)<br>0.0836(5)
Cr (8d)	Occ = 0.00	Occ = 0.50<br>0.2452(4)<br>0.1278(4)<br>0.0820(3)	Occ = 1.04<br>0.2444(4)<br>0.1290(3)<br>0.0821(3)	Occ = 1.50<br>0.2471(6)<br>0.1286(4)<br>0.0822(3)	Occ = 2.00<br>0.2454(5)<br>0.1280(4)<br>0.0822(3)	Occ = 2.67<br>0.2437(2)<br>0.1281(3)<br>0.0816(2)	Occ = 4.00<br>0.2549(7)<br>0.1287(7)<br>0.0835(4)	Occ = 5.33<br>0.2430(7)<br>0.1270(15)<br>0.0836(5
C (4c)	Occ = 4.00<br>0.0000<br>0.3640<br>0.2500	Occ = 4.00<br>0.0000<br>0.3640<br>0.2500	Occ = 4.00<br>0.0000<br>0.3640<br>0.2500	Occ = 4.00<br>0.0000<br>0.3640<br>0.2500	Occ = 4.00<br>0.0000<br>0.3640<br>0.2500	Occ = 4.00<br>0.0000<br>0.3640<br>0.2500	Occ = 4.00<br>0.0000<br>0.3640<br>0.2500	Occ = 4.00<br>0.0000<br>0.3640<br>0.2500
Phase fraction (wt%)	98	98	99	98	98	99	98	95
Global Chi^2	4.58	6.05	5.95	6.62	4.26	4.47	3.10	2.86

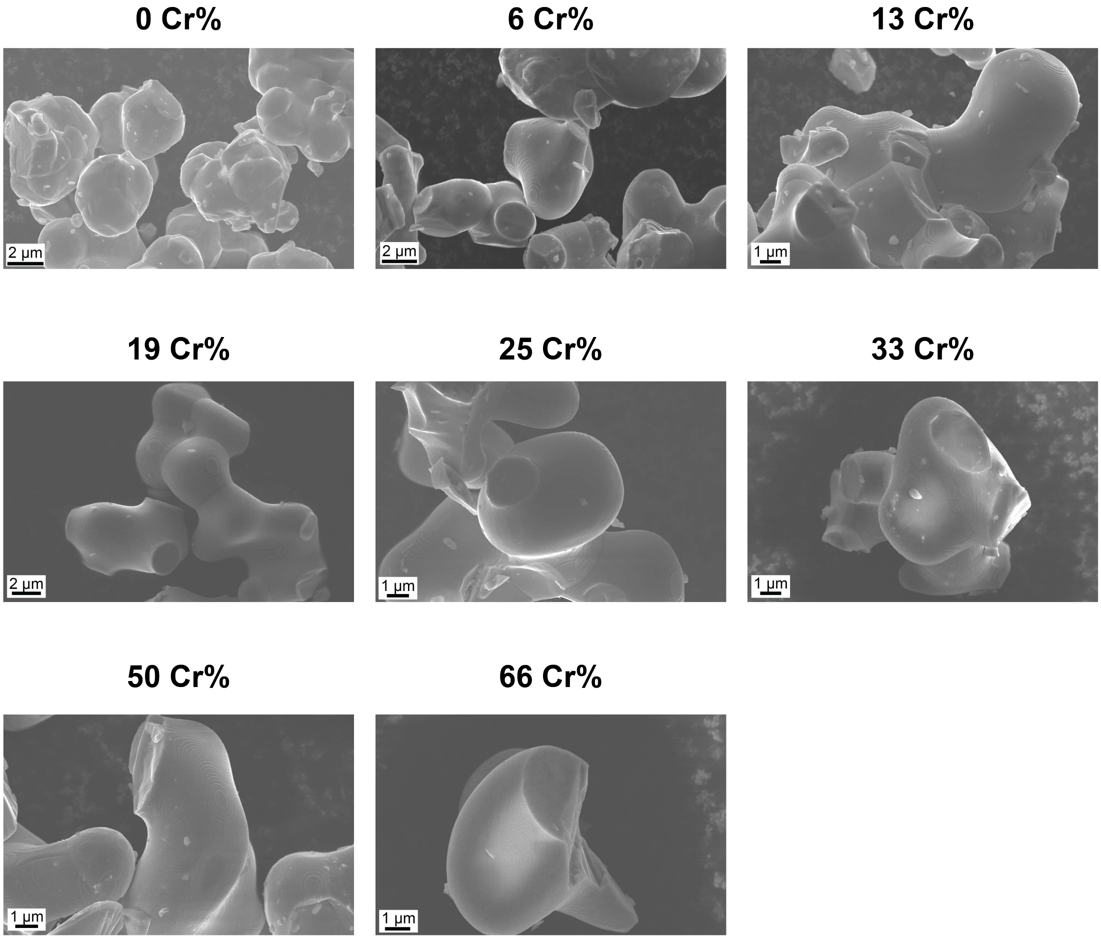

Figure S 6: SEM images $Mo_{2-x}Cr_xC$ with different Cr at.%.

Experimental Details II: 221-MAX Phase

Table S 9: Rietveld refinement summary of $Mo_{2-x}Cr_xGa_2C$, nominal Cr = 0%. The value in parentheses is the error (if not present, the parameter was not refined). The Cr occupancy was kept constant and not refined (see Experimental Details).

Phases	$Mo_{2-x}Cr_xGa_2C$	$Mo_{2-x}Cr_xC$	$Mo_{2-x}Cr_xGaC$
Space group	$P6_3/mmc$ (#194)	*Pbcn* (#60)	$P6_3/mmc$ (#194)
a (Å)	3.03657 (2)	4.73363	3.03910
b (Å)	3.03657 (2)	6.02887	3.03910
c (Å)	18.08703 (10)	5.20692	13.18252
α	90.000	90.000	90.000
β	90.000	90.000	90.000
γ	120.000	90.000	120.000
Mo	4f, Occ = 4.00<br>0.33333<br>0.66667<br>0.06548(4)	8d, Occ = 8.00<br>0.24900<br>0.12800<br>0.08270	4f, Occ = 4.00<br>0.33333<br>0.66667<br>0.58921
Ga	4f, Occ = 4.00<br>0.3333<br>0.66667<br>0.68243(6)		2c, Occ = 2.00<br>0.33333<br>0.66667<br>0.25000
C	2a, Occ = 2.00<br>0.0000<br>0.0000<br>0.0000	4c, Occ = 4.00<br>0.0000<br>0.3640<br>0.2500	2a, Occ = 2.00<br>0.0000<br>0.0000<br>0.0000
Pref Orientation	-0.488(0.004)<br>[002]	0.000<br>[001]	0.000<br>[001]
Wt%	99.33 (0.74)	0.55 (0.03)	0.12 (0.0)
Rietveld Information	R_p: 22.1 R_{wp}: 26.1 R_{exp}: 8.88 Global Chi^2: 10.9		

Table S 10: Rietveld refinement summary of $Mo_{2-x}Cr_xGa_2C$, nominal Cr = 6.25%. The value in parentheses is the error (if not present, the parameter was not refined). The Cr occupancy was kept constant and not refined (see Experimental Details).

Phases	$Mo_{2-x}Cr_xGa_2C$	$Mo_{2-x}Cr_xC$	$Mo_{2-x}Cr_xGaC$
Space group	*P6₃/mmc* (#194)	*Pbcn* (#60)	*P6₃/mmc* (#194)
a (Å)	3.03081(3)	4.72178	3.03615
b (Å)	3.03657(2)	6.01718	3.03615
c (Å)	18.05578(22)	5.17382	13.10706
α	90.000	90.000	90.000
β	90.000	90.000	90.000
γ	120.000	90.000	120.000
Mo	4f, Occ = 3.74<br>0.33333<br>0.66667<br>0.06341(4)	8d, Occ = 7.48<br>0.24150<br>0.12914<br>0.08183	4f, Occ = 3.74<br>0.33333<br>0.66667<br>0.59435
Cr	4f, Occ = 0.26<br>0.33333<br>0.66667<br>0.06341(4)	8d, Occ = 0.52<br>0.24150<br>0.12914<br>0.08183	4f, Occ = 0.26<br>0.33333<br>0.66667<br>0.59435
Ga	4f, Occ = 4.00<br>0.3333<br>0.66667<br>0.67940(4)		2c, Occ = 2.00<br>0.33333<br>0.66667<br>0.25000
C	2a, Occ = 2.00<br>0.0000<br>0.0000<br>0.0000	4c, Occ = 4.00<br>0.0000<br>0.3640<br>0.2500	2a, Occ = 2.00<br>0.0000<br>0.0000<br>0.0000
Pref Orientation	-0.413 (0.005)<br>[002]	0.76(0.10)<br>[001]	0.23(0.04)<br>[001]
Wt%	94.34 (0.87)	2.24 (0.35)	3.42 (0.20)
Rietveld Information	R_p: 27.5 R_{wp}: 29.3 R_{exp}: 9.62 Global Chi^2: 9.28		

Table S 11: Rietveld refinement summary of $Mo_{2-x}Cr_xGa_2C$, nominal Cr = 13%. The value in parentheses is the error (if not present, the parameter was not refined). The Cr occupancy was kept constant and not refined (see Experimental Details).

Phases	$Mo_{2-x}Cr_xGa_2C$	$Mo_{2-x}Cr_xC$	$Mo_{2-x}Cr_xGaC$
Space group	*P6₃/mmc* (#194)	*Pbcn* (#60)	*P6₃/mmc* (#194)
a (Å)	3.02724 (7)	4.69506(61)	3.02510(10)
b (Å)	3.02724 (7)	5.98356(78)	3.02510(10)
c (Å)	18.03406 (53)	5.22987(161)	13.11095(54)
α	90.000	90.000	90.000
β	90.000	90.000	90.000
γ	120.000	90.000	120.000
Mo	4f, Occ = 3.48<br>0.33333<br>0.66667<br>0.06491(13)	8d, Occ = 6.96<br>0.22238(109)<br>0.13923(62)<br>0.04117(378)	4f, Occ = 3.48<br>0.33333<br>0.66667<br>0.59075(16)
Cr	4f, Occ = 0.52<br>0.33333<br>0.66667<br>0.06491(13)	8d, Occ = 1.04<br>0.22238(109)<br>0.13923(62)<br>0.04117(378)	4f, Occ = 0.52<br>0.33333<br>0.66667<br>0.59075(16)
Ga	4f, Occ = 4.00<br>0.3333<br>0.66667<br>0.67940		2c, Occ = 2.00<br>0.33333<br>0.66667<br>0.25000
C	2a, Occ = 2.00<br>0.0000<br>0.0000<br>0.0000	4c, Occ = 4.00<br>0.0000<br>0.3640<br>0.2500	2a, Occ = 2.00<br>0.0000<br>0.0000<br>0.0000
Pref Orientation	0.093 (0.008)<br>[002]	0.99(0.03)<br>[001]	-0.24(0.01)<br>[001]
Wt%	38.82 (0.60)	4.32 (0.32)	56.86 (0.93)
Rietveld Information	R_p: 26.3 R_{wp}: 29.0	R_{exp}: 10.87	Global Chi^2: 8.34

Table S 12: Rietveld refinement summary of $Mo_{2-x}Cr_xGa_2C$, nominal Cr = 18.75%. The value in parentheses is the error (if not present, the parameter was not refined). The Cr occupancy was kept constant and not refined (see Experimental Details).

Phases	$Mo_{2-x}Cr_xGa_2C$	$Mo_{2-x}Cr_xC$	$Mo_{2-x}Cr_xGaC$
Space group	*P6₃/mmc* (#194)	*Pbcn* (#60)	*P6₃/mmc* (#194)
a (Å)	3.02506(14)	4.69075(29)	3.02157(4)
b (Å)	3.02506(14)	5.97817(54)	3.02157(4)
c (Å)	18.04472(152)	5.16416(45)	13.09089(24)
α	90.000	90.000	90.000
β	90.000	90.000	90.000
γ	120.000	90.000	120.000
Mo	4f, Occ = 3.25<br>0.33333<br>0.66667<br>0.06623(32)	8d, Occ = 6.50<br>0.24150<br>0.12914<br>0.08183	4f, Occ = 3.25<br>0.33333<br>0.66667<br>0.58951(8)
Cr	4f, Occ = 0.75<br>0.33333<br>0.66667<br>0.06623(32)	8d, Occ = 1.50<br>0.24900<br>0.12800<br>0.08270	4f, Occ = 0.75<br>0.33333<br>0.66667<br>0.58951(8)
Ga	4f, Occ = 4.00<br>0.3333<br>0.66667<br>0.67940		2c, Occ = 2.00<br>0.33333<br>0.66667<br>0.25000
C	2a, Occ = 2.00<br>0.0000<br>0.0000<br>0.0000	4c, Occ = 4.00<br>0.0000<br>0.3640<br>0.2500	2a, Occ = 2.00<br>0.0000<br>0.0000<br>0.0000
Pref Orientation	0.17(0.02)<br>[002]	0.00<br>[001]	-0.081 (0.005)<br>[001]
Wt%	9.64 (0.31)	2.75 (0.08)	87.61 (0.81)
Rietveld Information	R_p: 23.3 R_{wp}: 24.8	R_{exp}: 11.26	Global Chi^2: 6.29

Table S 13: Rietveld refinement summary of $Mo_{2-x}Cr_xGa_2C$, nominal Cr = 25%. The value in parentheses is the error (if not present, the parameter was not refined). The Cr occupancy was kept constant and not refined (see Experimental Details).

Phases	$Mo_{2-x}Cr_xGa_2C$	$Mo_{2-x}Cr_xC$	$Mo_{2-x}Cr_xGaC$
Space group	*P6₃/mmc* (#194)	*Pbcn* (#60)	*P6₃/mmc* (#194)
a (Å)	3.02401(22)	4.65620(59)	3.01516(5)
b (Å)	3.02401(22)	6.04573(118)	3.01516(5)
c (Å)	18.00070(212)	5.12083(78)	13.06311(26)
α	90.000	90.000	90.000
β	90.000	90.000	90.000
γ	120.000	90.000	120.000
Mo	4f, Occ = 3.00<br>0.33333<br>0.66667<br>0.06341	8d, Occ = 6.00<br>0.24150<br>0.12914<br>0.08183	4f, Occ = 3.00<br>0.33333<br>0.66667<br>0.59435(7)
Cr	4f, Occ = 1.00<br>0.33333<br>0.66667<br>0.06341	8d, Occ = 2.00<br>0.24150<br>0.12914<br>0.08183	4f, Occ = 1.00<br>0.33333<br>0.66667<br>0.59435(7)
Ga	4f, Occ = 4.00<br>0.3333<br>0.66667<br>0.67940		2c, Occ = 2.00<br>0.33333<br>0.66667<br>0.25000
C	2a, Occ = 2.00<br>0.0000<br>0.0000<br>0.0000	4c, Occ = 4.00<br>0.0000<br>0.3640<br>0.2500	2a, Occ = 2.00<br>0.0000<br>0.0000<br>0.0000
Pref Orientation	0.000<br>[002]	0.000<br>[002]	-0.016(0.004)<br>[001]
Wt%	0.63 (0.05)	0.50 (0.06)	98.87 (0.89)
Rietveld Information	R_p: 22.9 R_{wp}: 27.3 R_{exp}: 11.18 Global Chi^2: 7.81		

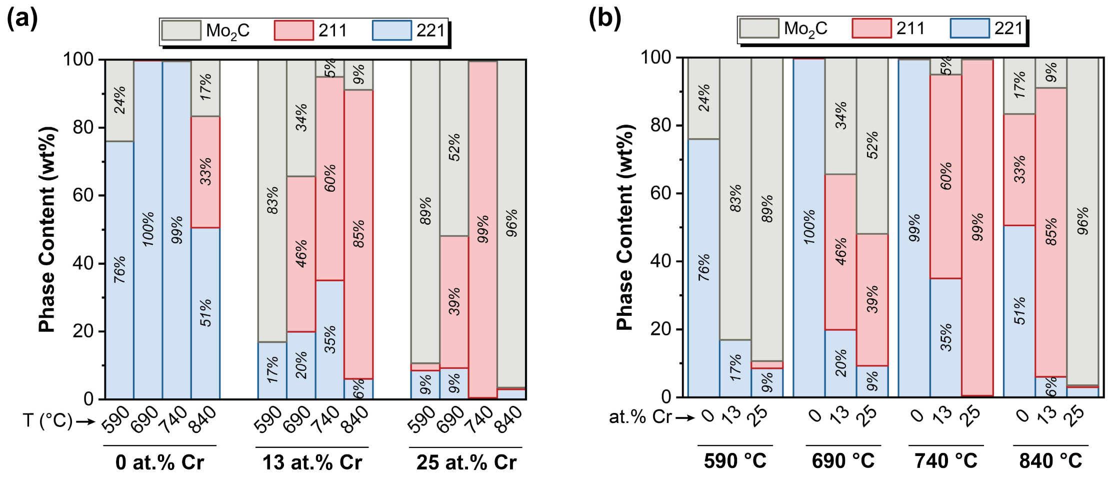

Figure S 7: Summary of phase contents estimated by Rietveld analysis for different synthesis conditions. (a) Effect of Cr alloying at different temperatures (b) Effect of different Cr content at the same temperature.

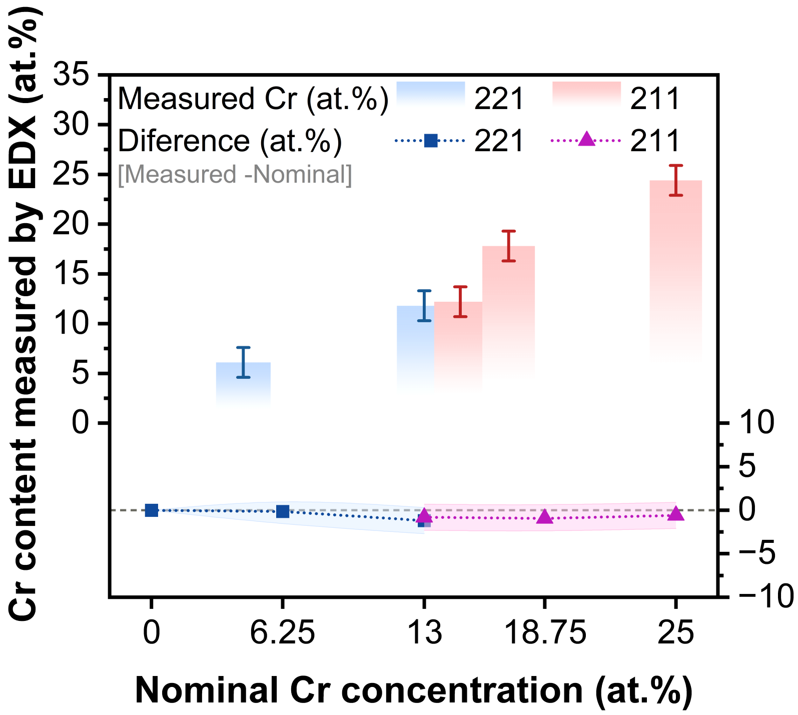

Figure S 8: Measured EDX Cr concentration (bars) for both 211 and 221-MAX phases vs nominal Cr contents. difference between the measured and nominal Cr concentration (dotted red line) with its associated statistical uncertainty (shaded region).

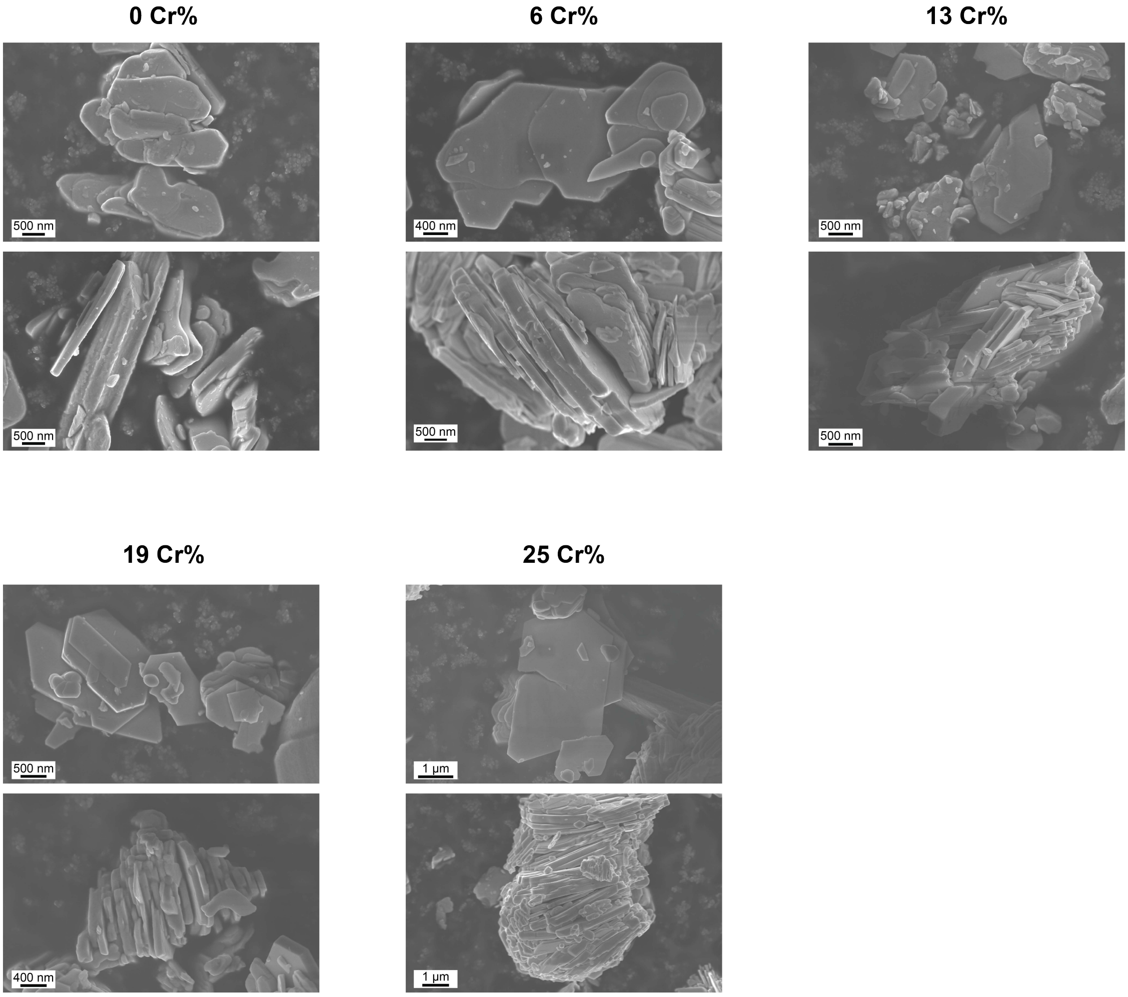

Figure S 9: SEM images of 221-MAX Phases with different Cr content.

Experimental Details III: $Mo_{2-x}CT_z$ MXenes

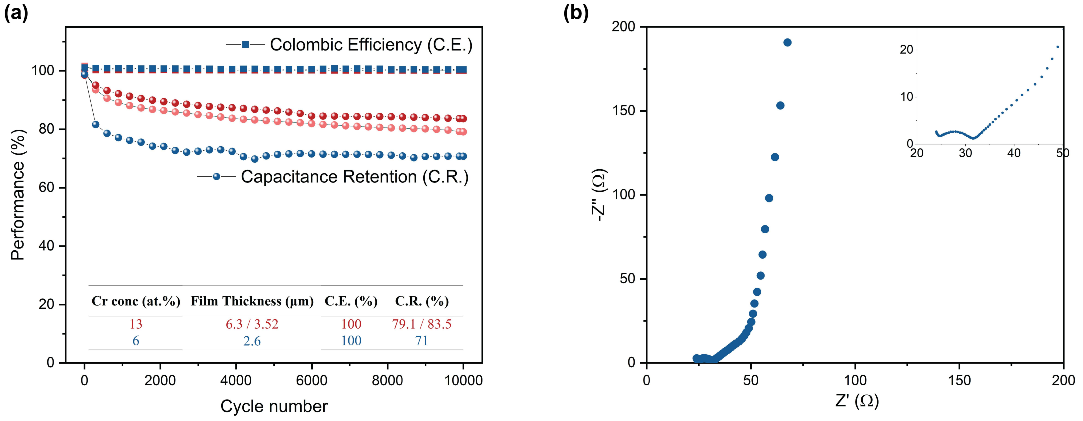

Figure S 10: Electrochemical characterization of a vacuum-filtered $Mo_{1.87}CT_z$ film (a) Cyclic stability at a current density of 10 A g^{-1}. (b) Nyquist plots, inset shows the high-frequency range.
